# A toxic gain-of-function variant in *MAPK8IP3* provides insights into JIP3 cellular roles

**DOI:** 10.1172/jci.insight.187199

**Published:** 2025-03-20

**Authors:** Wei Zhang, Swapnil Mittal, Ria Thomas, Anahid Foroughishafiei, Ricardo Nunes Bastos, Wendy K. Chung, Konstantina Skourti-Stathaki, Stanley T. Crooke

**Affiliations:** 1 n-Lorem Foundation, Carlsbad, California, USA.; 2 ONI Inc., San Diego, California, USA.; 3 Boston Children’s Hospital, Harvard Medical School, Brookline, Massachusetts, USA.

**Keywords:** Cell biology, Genetics, Therapeutics, Apoptosis, Genetic diseases, Neurodevelopment

## Abstract

Mitogen-activated protein kinase 8 interacting protein 3 (*MAPK8IP3*) gene encoding a protein called JIP3 is an adaption protein of the kinesin-1 complex known to play a role in axonal transport of cargo. Mutations in the gene have been linked to severe neurodevelopmental disorders, resulting in developmental delay, intellectual disability, ataxia, tremor, autism, seizures, and visual impairment. A patient who has a missense mutation in the *MAPK8IP3* gene (c. 1714 C>T, Arg578Cys) (R578C) manifests dystonia, gross motor delay, and developmental delay. Here, we showed that the mutation was a toxic gain-of-function mutation that altered the interactome of JIP3; disrupted axonal transport of late endosomes; increased signaling via c-Jun N-terminal kinase, resulting in apoptosis; and disrupted dopamine receptor 1 signaling while not affecting dopamine receptor 2 signaling. Furthermore, in the presence of the mutant protein, we showed that an 80% reduction of mutant JIP3 and a 60% reduction of WT JIP3 by non-allele-selective phosphorothioate-modified antisense oligonucleotides was well tolerated by several types of cells in vitro. Our study identifies what we believe to be several important new roles for JIP3 and provides important insights for therapeutic approaches, including antisense oligonucleotide reduction of JIP3.

## Abstract 2

JIP3 mutation R587C acts as a severe toxic gain of function and antisense oligonucleotides (ASOs) correct all proximal pathological phenotypes.

## Introduction

The mitogen-activated protein kinase 8 interacting protein 3 (*MAPK8IP3*) gene encodes the c-Jun N-terminal kinase–interacting (JNK-interacting) protein 3 (JIP3), which plays a pivotal role in cellular processes. Predominantly recognized for its involvement in lysosomal and JNK trafficking along the axonal cytoskeleton, the absence of JIP3 in neuronal cells results in the accumulation of these cargoes, leading to the disruption of the axonal cytoskeleton (1–4). The significance of JIP3 extends to its regulation of dynein, kinesin, and TrkB, further underscoring its role in cytoskeletal trafficking (5–7). However, a comprehensive understanding of its functions is incomplete. Notably, JIP3 has emerged as a crucial mediator of the JNK signaling pathway, challenging the perception that its major role is as a molecular chauffeur. Evidence suggests that the loss of JIP3 can rescue cardiac hypertrophy by suppressing JNK signaling (8). JIP3 operates not only as a scaffold for various MAPK proteins but also participates in the phosphorylation cascade, highlighting its multifaceted role in cellular signaling (9, 10). Moreover, the homologous JIP4 exhibits overlapping functions with JIP3 in lysosome transport, both in nonneuronal and neuronal cells, implying potential compensatory effects between the two proteins (11, 12). The complex interactions of JIP3 in regulating intracellular trafficking, MAPK/JNK pathways, and cell signaling pathways emphasize its multifunctional significance in cellular homeostasis. The compensatory potential of JIP4 further adds to the complexity of the dynamic roles played by these proteins in maintaining cellular integrity and function.

Deficiency of JIP3 disrupts axonal transport, leading to neuronal degeneration across both invertebrate and vertebrate species. Notably, novel variants in the *MAPK8IP3* gene, such as c.1364A > G (p.Glu455Gly) associated with Smith-Magenis-like syndrome and the de novo variant c.281A > G (p.Tyr94Cys) identified in a substantial cohort of patients with autism, underscore the gene’s relevance in neurodevelopment (13, 14). De novo variants in *MAPK8IP3* give rise to a spectrum of neurodevelopmental disabilities, characterized by global developmental delays, intellectual impairment, and movement disorder (14). The underlying mechanism of pathogenic mutations often involves a loss of JIP3’s role in endosomal trafficking along microtubules, leading to cargo accumulation in neurons, triggering cytotoxicity and eventual cell death. However, rare mutations in JIP3, exemplified by the missense mutation c.1732C > T (p.Arg578Cys) (R578C) (OMIM #618443), can activate the JNK pathway, culminating in cell death and apoptosis. Investigations of this mutation revealed severe global developmental delays, seizures, hypotonia, and spasticity. Evidence suggesting toxic gain-of-function (TGOF) effects arose when expression of JIP3 R578C in zebrafish posterior lateral line neurons caused abnormal varicosities in the axons like those reported in knockout mice (15). Similar variants in the *MAPK8IP3* gene have been implicated in directly causing comparable phenotypes (16). However, the intricate mechanisms underlying these cytotoxic effects remain poorly understood, representing a critical area for further research. Here, we sought to unravel these complexities in order to advance our understanding of neurodevelopmental disorders associated with *MAPK8IP3* mutations and to address molecular mechanisms to inform potential therapeutic interventions.

The goals of our study were to determine if the c 1714 C > T Arg578Cys missense mutation is a TGOF mutation, to define the proximal molecular pathological mechanisms, to better understand the many roles subserved by JIP3, and to lay the foundation for a potential therapeutic approach to this and other TGOF mutations in JIP3. We demonstrated that the human R578C variant is a severe TGOF and that total JIP3/JIP4 activity may be important to normal cell function and survival. The proximal molecular pathological consequences of this variant include alterations in the normal JIP3 interactome and altered subcellular localization. Even though JIP3 has previously been implicated in lysosomal activity and mobility, using early endosomes (EEs), late endosomes (LEs), and lysosome-specific markers, we demonstrated that JIP3 is essential for LE mobility along microtubules and this function is disrupted by the R578C variant. We also showed that the R578C variant substantially increases JNK signaling leading to apoptosis and, importantly, disrupts dopamine receptor 1 (D1) signaling. Finally, we showed that a non-allele-selective phosphorothioate–modified (PS-modified) antisense oligonucleotide (ASO) (referred to as PS-ASO) designed to activate RNase H1 that lowers mutant JIP3 (R578C) (MT-JIP3) by 80%–90% and WT JIP3 (WT-JIP3) by 60%–70% rescued all identified pathological phenotypes, suggesting that PS-ASOs, which would reduce only the MT-JIP3, may be attractive therapeutic options for this and other TGOFs in *MAPK8IP3*.

## Results

### MT-JIP3 induces JNK signaling–dependent apoptosis in HeLa cells, reducing cell growth, and JIP4 interacts with JIP3.

**Figure 1:**
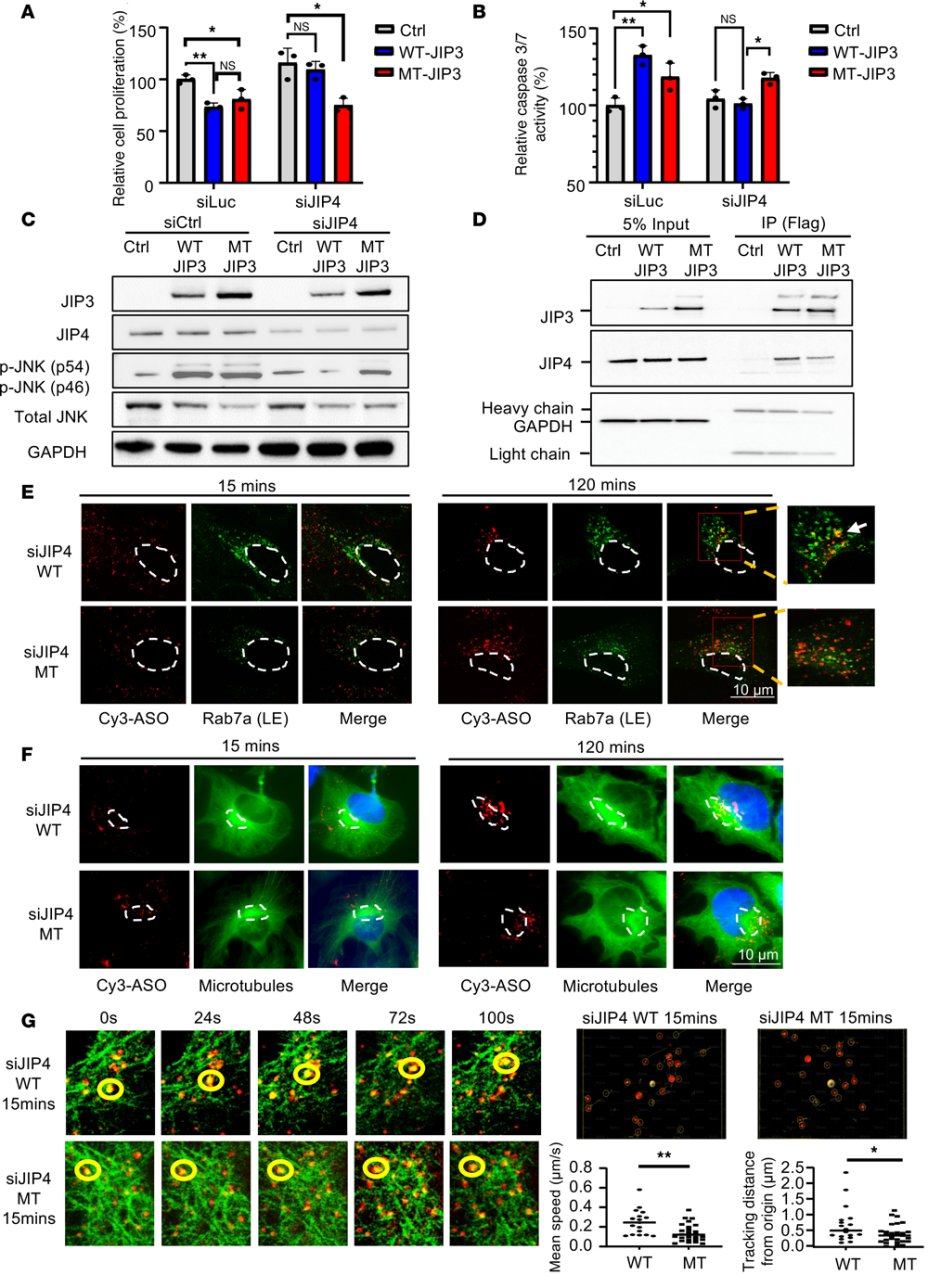
MT-JIP3 induces JNK signaling and disrupts cargo-laden LE mobility, thereby inhibiting HeLa cell growth. HeLa cells were transfected with siRNAs (siCtrl or siJIP4) recovered in fresh culture medium for 24 hours and then transfected overnight with plasmids encoding either Flag-tagged WT-JIP3 or MT-JIP3, followed by another 24-hour recovery before experiments. *P < 0.05, **P < 0.01. (**A**) Cell proliferation was measured by MTT assay. Data were normalized to cells transfected with empty vectors. *n* = 3. (**B**) Apoptosis was measured by caspase-3/7 activity. Data were normalized to cells transfected with empty vectors. *n* = 3. (**C**) Levels of p-JNK, p-c-Jun, JIP3, and JIP4 proteins were measured by Western blot. (**D**) JIP3 and JIP4 interaction was detected by co-IP. Briefly, Flag antibody was used for pulling down cell lysates, and JIP3 and JIP4 proteins were measured by Western blot. (**E**) Cell imaging of different siRNA/plasmid-treated HeLa cells incubated with 1 μM Cy3-labeled PS-ASO 446654 for different time points. The colocalization of Cy3-ASOs and Rab7 (LE [LE] marker) were observed by Keyence X800 microscope. Nuclei were marked with white dashed lines. Zoomed-in views were provided for the regions within the red frames, with white arrows indicating representative colocalization sites. Scale bar: 10 μm. (**F**) Trafficking of Cy3-ASOs along with microtubules in cells was detected using a Keyence BZ-X800 microscope. Microtubules were stained by anti-tubulin antibody. mTOCs were marked with white dashed lines. (**G**) Live-cell imaging of HeLa cells expressing GFP-labeled microtubules incubated with Cy3-ASO for 15 minutes. Images were recorded at different times, and snapshots are shown. The movement of a particular Cy3-ASO foci is marked by yellow cycles. Quantification of the speed and distances of Cy3-ASO foci to the original position, as measured from approximately 15 cells. *P* values were calculated with unpaired *t* test using Prism.

Since endogenous JIP3 protein was almost undetectable in HeLa cells (Figure 1C and Supplemental Figure 1A; supplemental material available online with this article; https://doi.org/10.1172/jci.insight.187199DS1), we first transiently transfected human WT-JIP3 and MT-JIP3 into HeLa cells. Considering the compensatory role of JIP4 (11), siRNAs were used to reduce the expression of JIP4 (Supplemental Figure 1A). Overexpression of WT-JIP3 or MT-JIP3 resulted in cell death and apoptosis (Figure 1, A and B). Importantly, when JIP4 expression was reduced, WT-JIP3 no longer induced cell death and apoptosis, in contrast to the persistent increase in cell death after expression of MT-JIP3 (Figure 1, A and B, and Supplemental Figure 1B), even when endogenous JIP4 was reduced. JIP3 has been reported to activate JNK signaling to induce apoptosis (9, 17). Overexpression of MT-JIP3 continued to activate JNK signaling when JIP4 was reduced (Figure 1C). The protective effect of JIP4 against JIP3’s cytotoxicity prompted us to explore whether they interact. Our findings revealed JIP4 in Flag immunoprecipitants of both Flag-tagged WT-JIP3 and MT-JIP3 in HeLa cells (Figure 1D). However, MT-JIP3 coprecipitated significantly less with JIP4 (Figure 1D and Supplemental Figure 1C) than with WT-JIP3. The apparent interaction of JIP3 and JIP4 suggests that heterodimers may be formed. Our data also suggest that excess JIP3/JIP4 activity may be cytotoxic and that MT-JIP3 may independently acquire cytotoxicity and result in less heterodimer formation.

### MT-JIP3 disrupts endosomal mobility following microtubule trafficking in HeLa cells and alters the binding affinity with associated proteins.

JIP3 has been reported to be an adaptor protein for endosomal trafficking (18). Endosomal processes are primarily responsible for cellular uptake and subcellular distribution of PS-ASOs and the process has been thoroughly studied (19). We examined PS-ASO as a cargo to follow endosomal movement after transient transfection of WT-JIP3/MT-JIP3 in HeLa cells with or without siRNA-mediated reduction of JIP4. Cy3-labeled PS-ASOs were incubated in HeLa cells for 15 minutes to visualize EE localization or for 120 minutes to visualize LE localization, as previous studies showed for ASO endosome trafficking (20). After the indicated time points, cells were stained for Rab7 (LE), which is a well-established LE marker. Colocalization analysis with Rab7 (LE marker) revealed similar patterns in WT-JIP3/MT-JIP3–expressing cells after 15 minutes (Figure 1E). However, at 2 hours, MT-JIP3 cells, particularly with reduction of JIP4, displayed reduced colocalization with Rab7 (LE marker) compared with WT-JIP3 cells (Figure 1E, Supplemental Figure 1D, and videos in Supplemental Figure 1F). Furthermore, Cy3-ASOs moved relatively rapidly along microtubules at the periphery of the cytoplasm toward the minus end (microtubule organizing center [mTOC] in the perinuclear area) in WT-JIP3 cells in 2 hours, as opposed to MT-JIP3 cells, in which reduced mTOC accumulation was observed (Figure 1F and Supplemental Figure 1E). This was further supported by the quantification of the mean trafficking speed and total trafficking distance of PS-ASO foci (Figure 1G). In addition, slower speed and shorter trafficking distance of total cellular PS-ASO foci were observed at 15 minutes after PS-ASO incubation in MT-JIP3 cells (Figure 1G), consistent with the observations that at 2 hours fewer PS-ASO foci accumulated at perinuclear areas (Figure 1F). Collectively, these results demonstrate that MT-JIP3 disrupts endosomal mobility along microtubules in HeLa cells.

### MT-JIP3 reduces cell growth and disrupts endosomal mobility in patient-derived fibroblasts.

**Figure 2:**
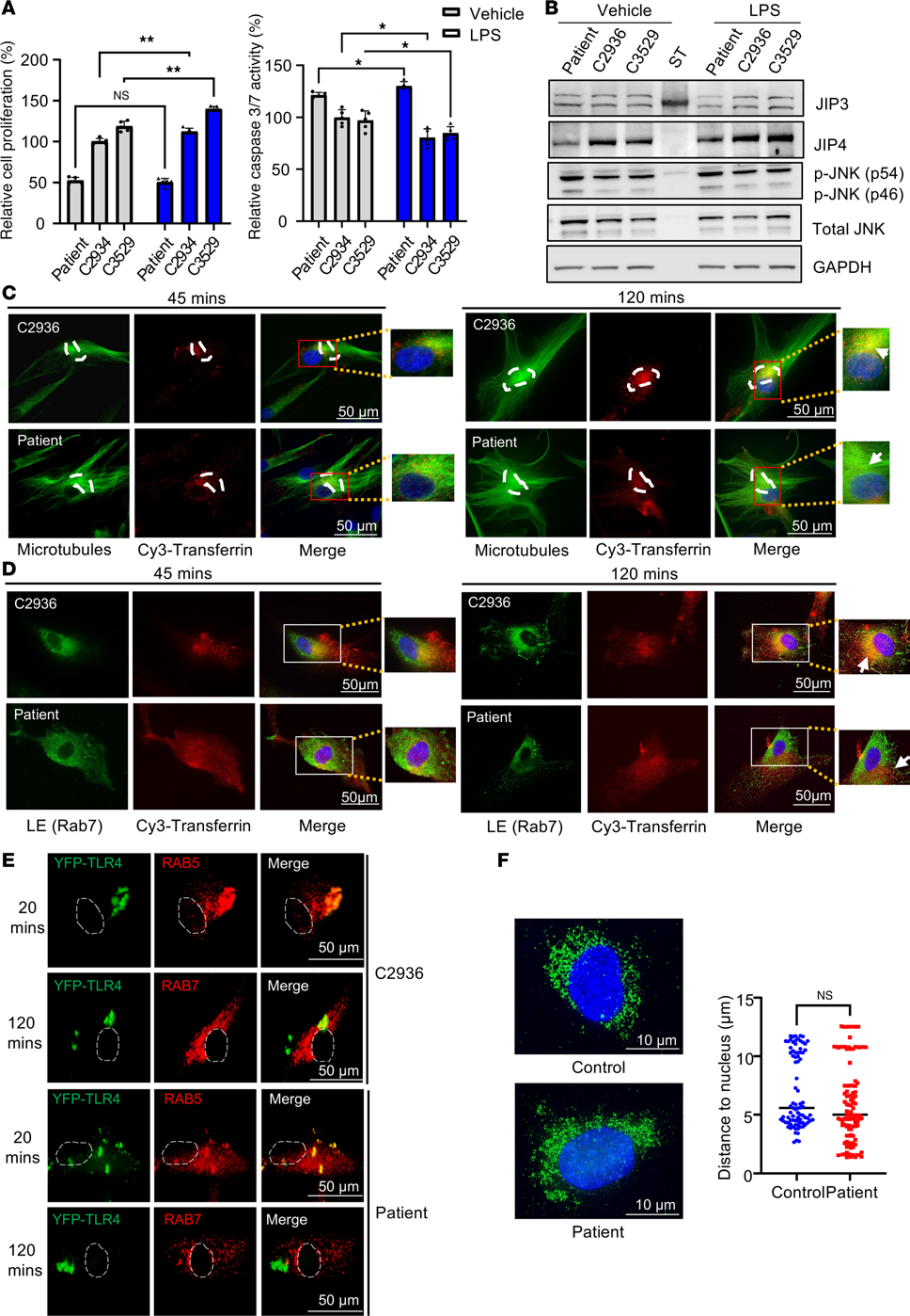
MT-JIP3 induces JNK signaling and disrupts cargo endosome mobility, thereby inhibiting cell growth in patient-derived fibroblasts. (**A**) Cells (2,000 cells/well) were seeded in 96 wells for 3 days. Cell proliferation was measured by MTT assay (left). Apoptosis induction was measured by caspase-3/7 activity (right). All of results were normalized to healthy patient-derived fibroblasts (control cells, 2,936). *n* = 5, *P < 0.05, **P < 0.01. (**B**) JIP3, JIP3, p-JNK, and total JNK proteins levels were measured by Western blot and normalized to GAPDH. (**C**) Trafficking of Cy3-transferrin along with microtubules in cells was detected with a Keyence microscope. Microtubules were stained by anti-tubulin antibody. mTOCs were marked with white dashed lines. Zoomed-in views were provided for the regions within the frames, with white arrows indicating representative colocalization sites. Scale bar: 50 μm; original magnification, ×63. (**D**) Cell imaging of either patient-derived fibroblasts or control fibroblasts incubated with 1 μM Cy3-transferrin for different time points. Colocalization of Cy3-transferrin and Rab7 (late endosome [LE] marker) was observed with a Keyence microscope. Zoomed-in views were provided for the regions within the frames, with white arrows indicating representative colocalization sites. Scale bar: 50 μm. (**E**) Cell imaging of either YFP-TLR4–transfected patient fibroblasts or healthy control fibroblasts incubated with 10 μg/mL LPS for different time points. Colocalization of activated YFP-TLR4 and Rab7 observed with a Keyence BZ-X800 microscope. Nuclei were marked with white dashed lines. Scale bar: 50 μm. (**F**) Cell imaging of lysosomes (LAMP1, a lysosome marker) either in patient-derived fibroblasts or healthy control fibroblasts observed by a Keyence microscope. Average distance between lysosome cluster to the center of nucleus in cells was measured by BZ-X800 analyzer software with the 3D model analysis function. Scale bar: 10 μm. Control cells, *n* = 85; patient cells, *n* =109. Unpaired *t* test.

To further substantiate our results in a patient-relevant cell system, we obtained skin fibroblasts from a patient carrying the *MAPK8IP3* R578C mutation. The patient-derived fibroblasts express MT-JIP3 at detectable levels (Figure 2B). Importantly, patient-derived fibroblasts showed less cell proliferation and higher induction of apoptosis than any of the other 3 human dermal control fibroblasts (control samples from healthy individuals were from Coriell Institute for Medical Research, GM02936, GM03529, and GM03440) (Figure 2A). Furthermore, LPS induced cell growth and reduced apoptosis in control cells, but patient cells did not respond to LPS (Figure 2A). The activation of p-JNK was elevated in patient fibroblasts compared with control fibroblasts in both the vehicle- and LPS-treated groups. Conversely, the basal level of JIP4 was lower in patient fibroblasts than in control fibroblasts (Figure 2B), which may explain why in this cell model reduction of JIP4 was not necessary to demonstrate the effects of MT-JIP3, as opposed to the HeLa cell model.

To further assess effects of MT-JIP3 on endosomal trafficking, we assessed Cy3-labeled transferrin endosomal trafficking and we observed less accumulation of transferrin in the mTOC region in patient fibroblasts after 120-minute incubation with labeled transferrin than that in control fibroblasts (Figure 2C). Moreover, we observed less colocalization of Cy3-labeled transferrin with Rab7 in patient fibroblasts than in control cells at 120-minute incubation (Figure 2D). To further confirm these observations with intracellular cargos, we transiently transfected cells with YFP-labeled TLR4 (21). YFP-labeled TLR4s were extensively colocalized with Rab7 in control fibroblasts upon LPS treatment for 2 hours, but very few colocalization foci were detected in patient fibroblasts (Figure 2E and Supplemental Figure 2B). However, the colocalization of TLR4 and Rab5 at 20 minutes showed no difference between control and patient-derived fibroblasts (Figure 2E and Supplemental Figure 2B). Furthermore, more colocalization of JIP3 with Rab5, but less colocalization with Rab7, was observed in the patient fibroblasts as compared with controls (Supplemental Figure 2, C and D). Furthermore, MT-JIP3 coprecipitated significantly less with JIP4 (Supplemental Figure 2E) than with WT-JIP3, and this was consistent with our observation in HeLa cells. It is important to note that we did not observe any changes in lysosomal location (Figure 2F), suggesting that the R578C mutation in MAPK8IP3 does not result in effects on lysosomes. This result is consistent with our data in the HeLa cell model, in which MT-JIP3 disrupted endosome mobility, pointing toward an established effect of MT-JIP3 on endosomal mobility, which is independent of the cell type. Together, these results demonstrate that MT-JIP3 reduces cell growth, enhances JNK signaling, and disrupts endosome mobility in patient fibroblasts. Thus, the R578C mutations appear to be cytotoxic via at least 2 mechanisms, increased JNK signaling and disruption of endosomal trafficking in two distinctly different types of cells.

### MT-JIP3 induces JNK signaling, reducing cell viability, and disrupts axonal trafficking in patient iPSC–derived neurons.

**Figure 3:**
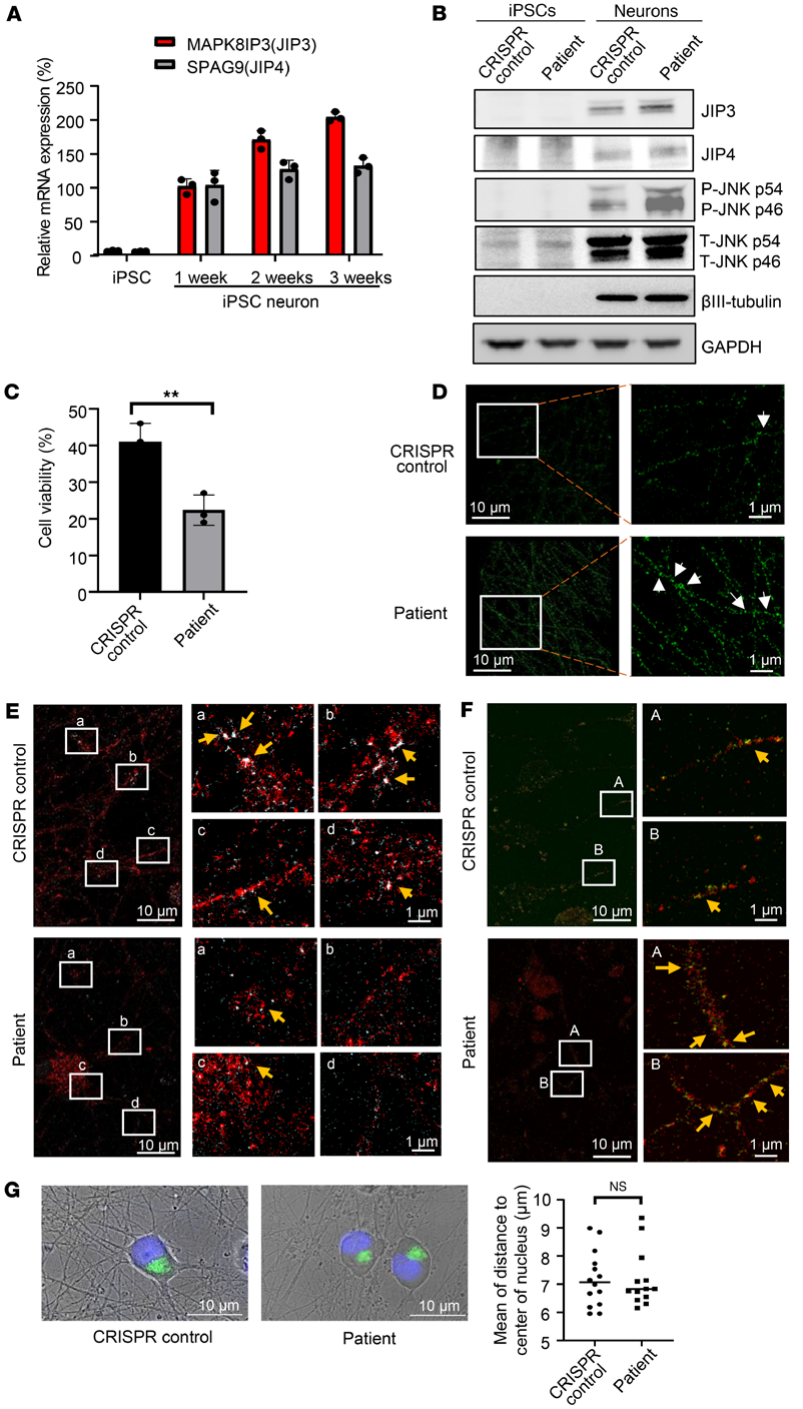
MT-JIP3 induces JNK signaling, disrupts cargo endosome mobility, and causes accumulation of viscosities, thereby increasing risk of death in patient iPSC–derived neurons. (**A**) mRNA levels of *MAPK8IP3* (JIP3) and *SPAG9* (JIP4) either in patient iPSCs or iPSC derived neurons were detected by qPCR. *n* =3. (**B**) JIP3, JIP4, p-JNK, total JNK, and βIII-tubulin protein levels either in CRISPR control or patient iPSC–derived neurons measured by Western blot. GAPDH was used as a loading control. (**C**) CRISPR control and patient iPSC–derived neurons cultured in 96 wells for 6 days after DIV14 (14 days in vitro). Cell culture medium was replaced every 2 days. Cell viability was measured by trypan blue cell count assay. *n* = 3, ***P < 0.001. (**D**) CRISPR control and patient iPSC–derived neurons were fixed and stained with anti-JIP3 antibody. Viscosities in long axons were detected by STORM with different magnifications. Representative viscosities were pointed out by white arrows. Scale bar: 10 μm (left); 1 μm (right). (**E**) Cell imaging of iPSC-derived neurons incubated with 1 μM Cy3-PS-ASO for 2 hours. Colocalization of Cy3-PS-ASO (light blue) and Rab7 (late endosome [LE] marker) (red) axons were observed by STORM on ONI Nanoimager. Representative colocalization sites (white) were pointed out by yellow arrows. Scale bar: 10 μm (left); 1 μm (right). (**F**) Colocalization of JIP3 and KIF5B either in CRISPR control or patient iPSC–derived neurons observed by STORM. Representative colocalization sites (yellow) are indicated by yellow arrows. Scale bar: 10 μm (left); 1 μm (right). (**G**) Cell imaging of lysosomes (LAMP1, a lysosome marker) either in CRISPR control or patient iPSC–derived neurons observed with a Keyence BZ-X800 microscope. The average distance between lysosome clusters to the center of nucleus in cells was measured by BZ-X800 analyzer software with the 3D model analysis function. Scale bar: 10 μm. CRISPR iPSC–derived neurons, *n* = 14; patient iPSC–derived neurons, *n* = 13. Unpaired *t* test.

To further dissect the role of MT-JIP3, we differentiated the patient’s induced pluripotent stem cells (iPSCs) cells into neuronal cells. The expression of *MAPK8IP3* (JIP3) RNA increased markedly as differentiation progressed. However, that was not the case for *SPAG9* (JIP4) RNA, and *SPAG9* (JIP4) expression only increased during differentiation from initial iPSC cells into neural cells (Figure 3A). This is consistent with our prediction that JIP4 expression is lower in neuronal cells and there is not enough compensation to maintain the normal functions of JIP3/JIP4 in the patient neurons. We also showed that the expression of JIP3 and JIP4 proteins was consistent with RNA expression. In patient-derived neuronal cells, therefore, JIP3 was well expressed, but JIP4 expression was relatively low (Figure 3B). We then assessed JNK signaling in patient neuronal cells. Our data show that JNK signaling was enhanced in patient-derived neuronal cells compared with control neurons (Figure 3B). Next, we assessed cell viability. Consistent with results in other cell types, the patient’s neuronal cells were less viable than control neurons (Figure 3C).

Varicosities refer to enlarged presynaptic boutons along the length of axon, which have a key function in neurotransmitter release and indicate the location of synapses under normal physiological conditions (22). We observed that more varicosities accumulated in axons of patient iPSC–derived neuronal cells by either stochastic optical reconstruction microscopy (STORM), which measures single molecule colocalization (Figure 3D and Supplemental Figure 3A), or Keyence microscopy (Supplemental Figure 3B). We also observed less of the colocalization of Cy3-PS-ASO (light blue) and Rab7 (red) in the axon and soma of the patient neuronal cells (Figure 3E). Interestingly, more JIP3 and Rab5 colocalization was shown in patient iPSC–derived neurons compared with that in CRISPR control cells (Supplemental Figure 3C). This suggests that patient iPSC–derived cells exhibit delayed endosomal movement. Furthermore, greater JIP3 (green) and KIF5b (red) colocalization was observed in patient iPSC–derived neurons compared with that in CRISPR controls (Figure 3F). This is consistent with our co-IP results showing altered binding of JIP3 and KIF5b in iPSC-derived neurons (Supplemental Figure 3D). Similar to that in patient-derived fibroblasts, we also did not observe any changes in lysosome distribution in the patient-derived neuronal cells (Figure 3G), further substantiating that the R578C mutation was not affecting lysosomes but late endosomes (LEs). These results indicate that MT-JIP3 impairs axonal trafficking, primarily of LEs in patient neuronal cells, and are consistent with our observations in other cell models that MT-JIP3 is a severely TGOF mutation that causes increased cell death via several mechanisms.

### Patient-derived fibroblasts and iPSC-derived neurons showed impaired D1 signaling.

**Figure 4:**
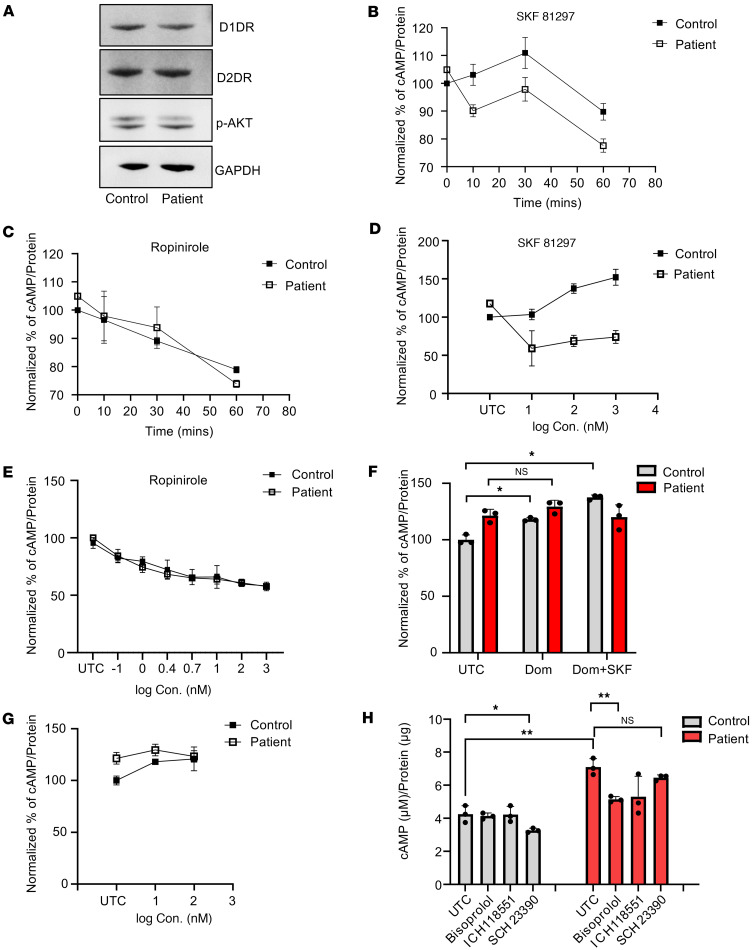
Dopamine signaling was impaired in patient fibroblasts (GM03440). (**A**) Dopamine receptor 1 and 2 (D1 and D2) and p-AKT proteins levels in fibroblasts measured by Western blot. GAPDH was used as a loading control. Either healthy fibroblasts or patient fibroblasts were seeded in 96 wells (10,000 cells/well) and treated with 1 μM (**B**) D1-selective agonist SKF 81297 and (**C**) D2-selective agonist ropinirole for different time points. cAMP levels were measured with a cAMP-Glo Assay (Promega). Data were normalized to untreated control (UTC) of cells at time 0. *n* = 3. Either healthy individual–derived fibroblasts or patient-derived fibroblasts were seeded in 96 wells (8,000 cells/well) and treated by different concentrations of (**D**) D1-selective agonist SKF 81297 and (**E**) D2-selective agonist ropinirole for 30 minutes. cAMP levels were measured by cAMP-Glo Assay (Promega). Data were normalized to UTC of cells. *n* = 3. (**F**) Fibroblasts were treated with 1 μM D2-selective antagonist domperidone (Dom) for 5 minutes before addition of 1 μM D1-selective agonist SKF 81297 (SKF) for 30 minutes. Data were normalized to UTC of cells. *n* = 3. *P < 0.05. (**G**) Fibroblasts were treated with different concentrations of D2-selective antagonist domperidone (Dom) for 30 minutes. cAMP levels were measured by cAMP-Glo Assay (Promega). Data were normalized to UTC of cells. *n* = 3. (**H**) Fibroblasts were treated with 1 μM β1-AR–selective antagonist bisoprolol, β2-AR–selective antagonist ICH118551, and D1-selective antagonist SCH23390 for 30 minutes. cAMP levels were measured by cAMP-Glo Assay (Promega). Data were normalized to UTC of cells. *n* = 3. *P < 0.05, **P < 0.01.

NLR family pyrin domain containing 2 (*NLRP2*) has been reported to be upregulated in iPSCs in bipolar disorder–associated (BD-associated) inflammation (23). Furthermore, iPSC-derived BD cortical neural stem cells harbor multiple abnormalities in the dopamine and GABA receptor pathways. Much higher transcript levels of *NLRP2* were observed in patient iPSC–derived neurons than in CRISPR controls (Supplemental Figure 4A). This prompted us to consider whether the mutation affects dopamine signaling, which is closely associated with Parkinson’s disease and might contribute to the limited movement observed in the patient (24). Given that we have employed patient skin fibroblasts for our experiments, and they proved a very important cell system in our efforts to understand the effects of the R578C mutation and develop potential therapeutic approaches, we examined the expression of dopamine receptors in fibroblasts. Dopamine receptors were abundantly expressed in both healthy and patient-derived fibroblasts indeed (Figure 4A). This is in line with previous studies that have highlighted the expression of these receptors in fibroblasts (25). Furthermore, the downstream signal, p-AKT, of dopamine receptor 2 (D2) receptors was activated (Figure 4A). This result demonstrates that fibroblasts can be used as a cell model to detect the effects of mutations on dopamine signaling. Next, we used D1- and D2-selective agonists, SKF81297 (SKF) and ropinirole (ROP), to activate D1 and D2, respectively (26, 27). It is evident that cAMP in control cells increased markedly after 30 minutes of SKF treatment (Figure 4B). In contrast, there was no significant increase in cAMP in patient-derived fibroblasts (Figure 4B). However, upon ROP treatment, cAMP was greatly reduced in healthy control- and patient-derived fibroblasts, and this was time dependent (Figure 4C). D1 and D2 signaling in control cells was a dose dependent (Figure 4, D and E), while only dose-dependent D2 signaling was observed in patient-derived fibroblasts (Figure 4E). These results indicate that D1 is coupled to cAMP signaling in control cells, as expected, but not in patient-derived fibroblasts. In contrast, D2 coupling was apparent in both control- and patient-derived fibroblasts. To further confirm this finding, we used the D2-selective antagonist, domperidone (Dom), to inhibit the effect of D2 and then used SKF to activate D1 in both cells. We found that only control cells responded to D1 activation (Figure 4F). This further suggests that D1 signaling is uncoupled in patient-derived fibroblasts. Although the basal level of cAMP in patient cells was higher than that in control cells (Supplemental Figure 4B), the cAMP in patient cells was unchanged when D2 activation was blocked by the D2-selective antagonist (Dom) (Figure 4G). These results suggest that the higher basal level in patient cells may be driven by other surface G proteins. To further address this question, β1 and β2 adrenergic receptor–selective (AR-selective) antagonists (bisoprolol and ICH118551) were used to block AR-induced cAMP production in both cells (28–30). Interestingly, the basal level of cAMP in patient cells was dramatically decreased and restored to the level of control cells, but D1 antagonist (SCH23390) only inhibited the cAMP induction in control cells (Figure 4H). This further demonstrates that the higher basal level of cAMP in patient cells is likely driven by AR receptors.

**Figure 5:**
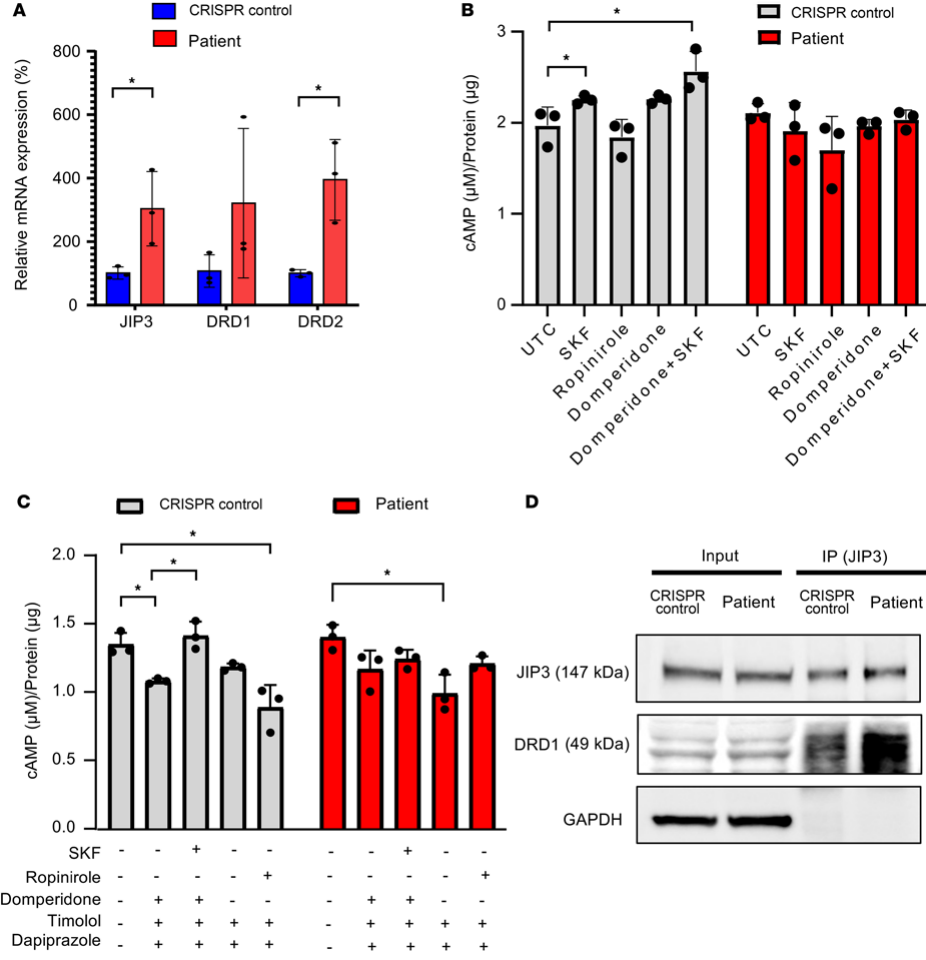
Dopamine signaling was uncoupled in patient iPSC–derived neurons. (**A**) mRNA levels of *MAPK8IP3* (JIP3), *DRD1*, and *DRD2* either in CRISPR control or patient iPSC–derived neurons were measured by qPCR. *n* = 3. *P < 0.05. (**B**) iPSC-derived neurons were treated with 1 μM D1-selective agonist SKF 81297, D2-selective agonist ropinirole, and 1 μM D2 selective antagonist domperidone for 30 minutes. cAMP levels were measured by cAMP-Glo Assay (Promega). Data were normalized to UTC of cells. *n* = 3. *P < 0.05. (**C**) iPSC-derived neurons were treated with 1 μM D2-selective antagonist domperidone and/or pan–β-AR antagonist timolol and/or pan–α-AR antagonist dapiprazole for 5 minutes before addition of 1 μM D1-selective agonist SKF 81297 or D2-selective agonist ropinirole for 30 minutes. cAMP levels were measured by cAMP-Glo Assay (Promega). Data were normalized to UTC of cells. *n* = 3. *P < 0.05. (**D**) The interaction of JIP3 and D1 was detected by co-IP. Briefly, JIP3 antibody was used for pulling down cell lysates and D1 and JIP3 proteins were measured by Western blot with D1 and JIP3 antibodies, respectively.

To further confirm the defect in D1 signaling in the most disease-relevant patient cells, we repeated the experiments in patient iPSC–derived neuronal cells. Although D1 and D2 receptors were more highly expressed in patient iPSC–derived neuronal cells as compared with the CRISPR control neurons (Figure 5A), cAMP in patient cells was not induced by SKF, irrespective of the presence or absence of D2 activation (Figure 5B). To further confirm this observation, we used β1 and β2 pan-AR antagonists (timolol and dapiprazole) to inhibit the effects of ARs on cAMP to selectively observe dopamine receptors activation (29, 31). As shown, it is evident that SKF still only activates D1 in control cells (Figure 5C). Next, to further substantiate the connection of JIP3 and D1 and understand the underlying mechanism of MT-JIP3–induced D1 uncoupling in patient cells, we employed co-IP to investigate the direct interaction between JIP3 and the dopamine receptor. As shown, D1 interacted with JIP3 and MT-JIP3 increased this interaction (Figure 5D). This result indicates that MT-JIP3 may overinteract with D1 to interrupt D1 signaling activation in patient cells. In summary, these results demonstrate that MT-JIP3 abnormally interacts with D1 to disrupt D1 signaling.

### Non-allele-selective PS 2′-methoxyethyl gapmer ASOs activate RNase H1–mediated reduction of both MT- and WT-JIP3 mRNA and protein, restore endosome mobility, and rescue patient fibroblasts from mutation-induced cell death.

**Figure 6:**
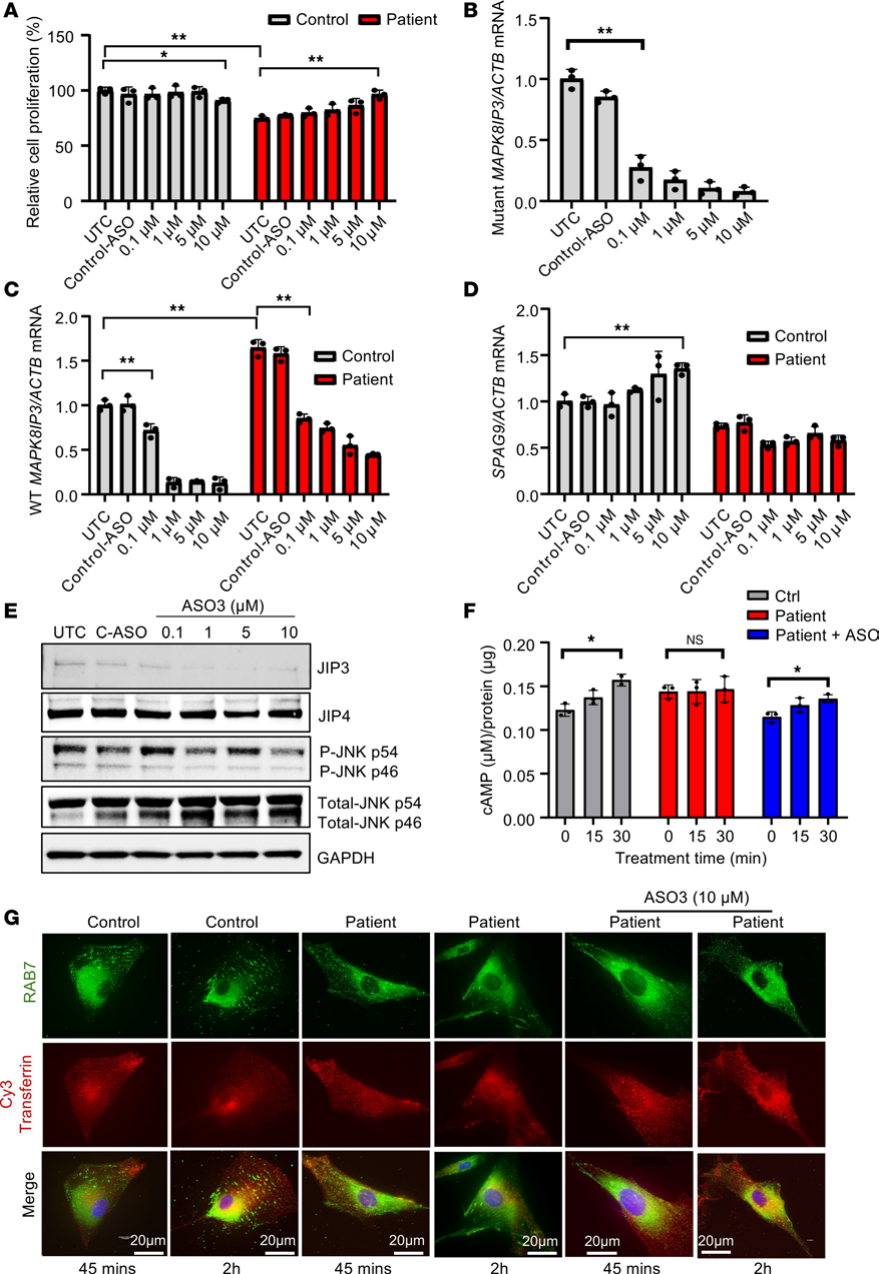
Non-allele-selective PS-ASOs rescue patient fibroblasts from MT-JIP3–induced cell toxicity. Healthy individual–derived (control) fibroblasts from GM03440 and patient-derived fibroblasts (5,000 cells/well) were electroporated with different concentrations of non-allele-selective ASO3 targeting *MAPK8IP3* (JIP3) and incubated in 96 wells with normal culture medium for different time points for different experiments. Scrabble PS-ASO 676630 (5 μM) was used as a control ASO. (**A**) After 72-hour incubation, cell proliferations were measured by MTT assay and normalized to UTC of control cells. *n* = 3, *P < 0.05, **P < 0.01. (**B**) After 48-hour incubation, RNA was extracted by GITC assay, and MT-MAPK8IP3 mRNA levels were measured using q-PCR. Data were normalized to UTC of control cells. *n* = 3, **P < 0.01. (**C**) WT *MAPK8IP3* mRNA levels were measured using q-PCR. Data were normalized to UTC of control cells. *n* = 3, **P < 0.01. (**D**) SPAG9 (JIP4) mRNA levels were measured using q-PCR. Data were normalized to UTC of control cells. *n* = 3, **P < 0.01. (**E**) Cell proteins were harvested after 72-hour incubation of ASOs with different concentrations. JIP3, JIP4, p-JNK, and total JNK were measured by Western blot. GAPDH was used as a loading control. (**F**) 10 μM non-allele-selective ASO3 was used to treat mutations in patient iPSC neurons for 72 hours, and then 1 μM D1 agonist (SKF 81297) was added to each test group with different incubation time points. cAMP levels were measured by cAMP-Glo Assay (Promega). Data were normalized to UTC of cells. *n* = 3, *P < 0.05. (**G**) After 48-hour ASO treatment, cell imaging of either patient fibroblasts or healthy control fibroblasts incubated with 1 μM Cy3-transferrin for different time points. Colocalization of Cy3-transferrin and Rab7 (late endosome [LE] marker) was observed with a Keyence BZ-X800 microscope. Scale bar: 20 μm.

While JIP4 can compensate for the loss of JIP3, our studies have shown that JIP4 levels were significantly reduced in patient-derived neuronal cells, limiting its compensatory potential. This suggests that an excessive reduction of WT-JIP3 might be toxic in neuronal cells. To assess the risk of reducing WT-JIP3 and determine the necessary degree of allele selectivity for ASOs designed to target only MT-JIP3, we synthesized non-allele-selective PS 2′-methoxyethyl gapmer ASOs (PS-2′MOE ASOs) that target both alleles of *MAPK8IP3*. PS-2’MOE ASOs are chemically modified oligonucleotides that bind to complementary mRNA sequences, forming DNA-RNA hybrids. This triggers RNase H1 to degrade the RNA strand, reducing the target mRNA levels. The PS-2′MOE modifications enhance stability and binding affinity, making them effective for gene silencing. These ASOs target both WT and mutant alleles, offering potential therapeutic benefits in conditions involving TGOF mutations. However, their non-allele selectivity can also pose risks by reducing essential proteins from WT alleles. Key advantages include enhanced stability and specificity, but challenges like off-target effects and effective delivery remain critical considerations. We developed and tested 3 PS-2′MOE ASOs (ASO1, ASO2, and ASO3, as listed in Supplemental Table 2) to target MAPK8IP3 (JIP3). These PS-ASOs successfully rescued cell proliferation in patient fibroblasts and suppressed *MAPK8IP3* expression without affecting *SPAG9* (JIP4) expression (Supplemental Figure 5, A and B). To confirm the TGOF of MT-JIP3, we used the non-allele-selective ASO3 to inhibit MT-JIP3 expression. This inhibition was dose dependent and significantly reduced MT-JIP3 expression in patient cells (Figure 6B). WT-JIP3 expression was also reduced in both control and patient cells, as expected due to the non-allele-selective nature of the ASO (Figure 6C). However, *SPAG9* (JIP4) expression was not affected (Figure 6D), indicating the specificity of the ASO for *MAPK8IP3* (JIP3).

When MT-JIP3 expression was inhibited, patient cell proliferation increased in a dose-dependent manner (Figure 6A), a result corroborated by applying ASO1 and ASO2 (Supplemental Figure 5C). This confirmed that MT-JIP3 contributes to reduced cell proliferation. Additionally, JNK signaling activation was attenuated by the ASO, further demonstrating the TGOF nature of MT-JIP3 (Figure 6E). We also observed that ASO treatment normalized endosomal movement; Cy3-transferrin in patient cells accumulated in the late endosomal area at 2 hours and colocalized with Rab7, similar to control cells (Figure 6G and Supplemental Figure 5D). Moreover, ASO3-treated patient-derived fibroblasts showed increased cAMP levels upon stimulation with the D1 agonist SKF (Figure 6F). These findings highlight the therapeutic potential of non-allele-selective ASOs in restoring normal cellular function in patient cells.

### Suppression of MT-JIP3 by ASOs rescues patient iPSC–derived neurons from mutation-induced cytotoxicity.

**Figure 7:**
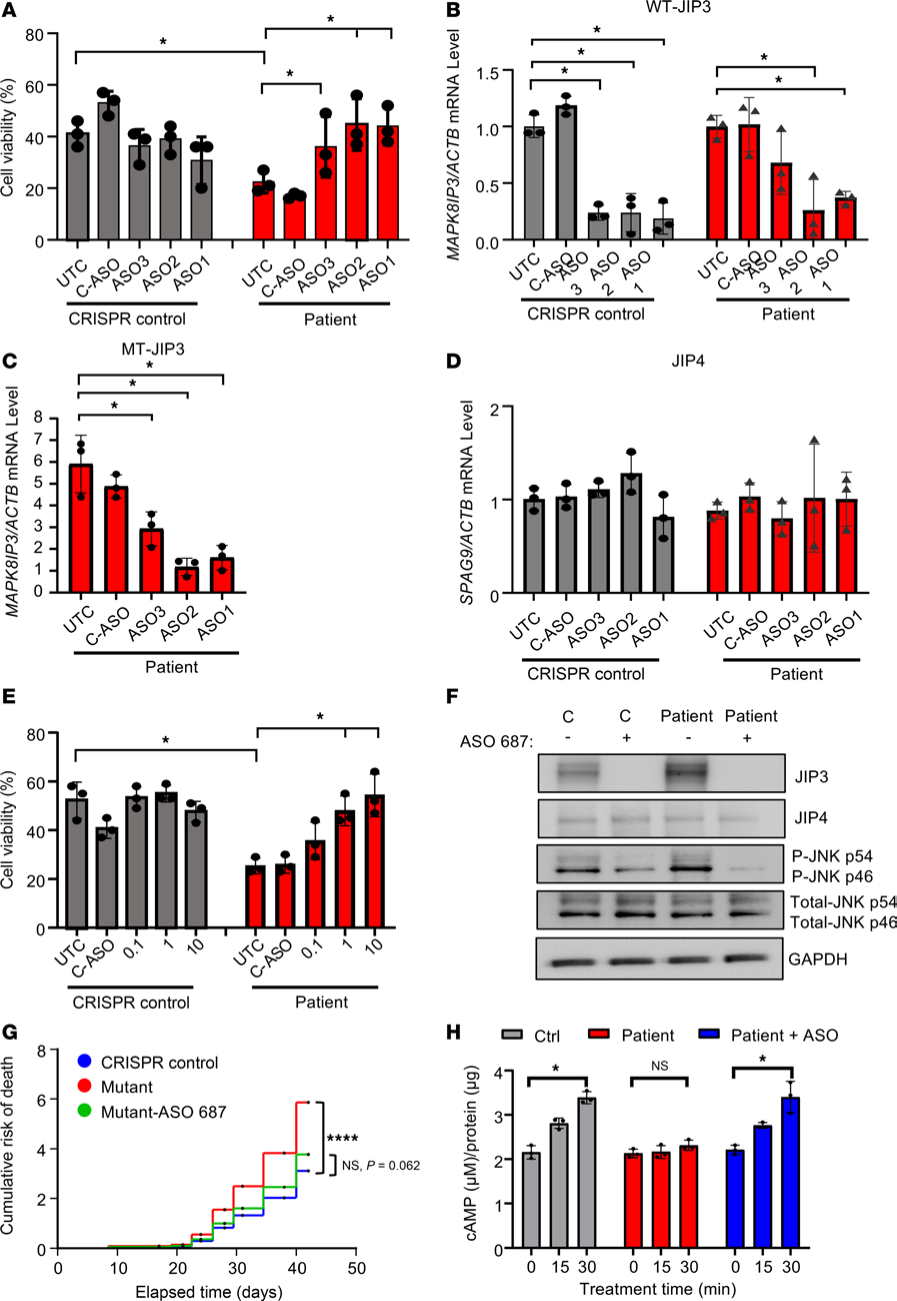
Non-allele-selective PS-ASOs rescue patient iPSC–derived neurons from MT-JIP3–induced cell toxicity. CRISPR control and patient iPSC–derived neurons were treated with 3 different non-allele-selective ASOs, including ASO1 (1713794), ASO2 (1713687), and ASO3 (1713510) targeting *MAPK8IP3* (JIP3) for 4 days. Scrabble PS-ASO 676630 was used as a control ASO. (**A**) Cell viability was measured by trypan blue cell count assay and normalized to UTC of CRISPR control cells. *n* = 3, *P < 0.05. (**B**) RNA was extracted by GITC assay, and MT-MAPK8IP3 mRNA level was measured using q-PCR. Data were normalized to UTC of control cells. *n* = 3, *P < 0.05. (**C**) WT-*MAPK8IP3* mRNA level was measured using q-PCR. Data were normalized to UTC of control cells. *n* = 3, *P < 0.05. (**D**) *SPAG9* (JIP4) mRNA levels were measured using q-PCR. Data were normalized to UTC of control cells. *n* = 3. (**E**) CRISPR control and patient iPSC–derived neurons were treated with different concentrations of non-allele-selective ASO2 targeting *MAPK8IP3* (JIP3) for 4 days. Scrabble PS-ASO 676630 was used as a control ASO. Cell viability was measured by trypan blue cell count assay and normalized to UTC of CRISPR control cells. *n* = 3, *P < 0.05. (**F**) Cell proteins were harvested after 4-day incubation of ASO2. JIP3, JIP4, and p-JNK were measured by Western blot. GAPDH was used as a loading control. (**G**) Expression of the MT-JIP3 in patient iPSC–derived neurons leads to increased risk of death compared with CRISPR control iPSC-derived neurons. ASO2 (5 μM) was used to treat mutation. Cells were screened using a Keyence microscope every 3 or 4 days. Viable cell numbers were manually counted in 3 representative areas from each well (center, upper center, and lower center). Cumulative risk was calculated by Cox proportional hazard regression in Prism 9.5. ****P < 0.0001. (**H**) 5 μM non-allele-selective ASO2 was used to treat mutations in patient iPSC neurons for 72 hours, and then 1 μM D1 agonist (SKF 81297) was added to each test group with different incubation time points. cAMP levels were measured using a cAMP-Glo Assay (Promega). Data were normalized to UTC of cells. *n* = 3. *P < 0.05.

Next, to substantiate the therapeutic potential of ASOs, we chose to employ the same 3 ASOs for the rescue experiments. All ASOs restored cell viability of patient iPSC–derived neurons to the level of CRISPR control neurons (Figure 7A). Furthermore, all 3 ASOs could effectively reduce the expression of MT-JIP3 mRNA, but no significant JIP4 reduction was observed (Figure 7, C and D), demonstrating their specificity. Among them, ASO2 was the most efficacious in reducing the expression of MT-JIP3 mRNA over 80%. It is worth noting that these ASOs are non-allele selective, thus the expression of WT-JIP3 mRNA was also reduced (Figure 7B). Importantly, WT-JIP3 was also reduced, but this was tolerated by the cells. In addition, when the expression of MT-JIP3 was inhibited by ASOs, cell viability increased significantly (Figure 7A). These data will provide substantial information toward a therapeutic avenue and subsequent ASO drug discovery and development, as they show that a lower allele selectivity can be tolerated for this gene target.

Next, we selected ASO2, which had the best efficacy, to conduct the dose-dependent experiment. The results showed that the ability of ASO2 to restore cell viability was dose dependent (Figure 7E). Furthermore, Western blot results showed that protein expression of JIP3 was significantly reduced upon cell treatment with ASO2, and the activation of JNK was also markedly weakened (Figure 7F). Cox proportional hazards analysis has been widely used to study the toxicological properties of a variety of substances associated with neurodegenerative diseases (32, 33); it generates a hazard ratio to represent relative and estimates risk of death for each group of iPSC-derived neurons. MT-JIP3 increased the hazard ratio in patient iPSC–derived neurons compared with WT-JIP3 in CRISPR control neurons, but ASO2 significantly reduced the MT-JIP3–induced hazard ratio (Figure 7G and Supplemental Figure 6). Additionally, ASO2 restored D1 coupling on patient iPSC–derived neurons in response to D1 agonist SKF stimulation (Figure 7H). These results collectively demonstrate that a non-allele-selective ASO rescues the patient iPSC–derived neurons from cytotoxicity.

## Discussion

Our results clarify the roles of WT-JIP3, the interactions of JIP3 and JIP4, the reductant functions of JIP3 and JIP4, and aspects of JIP3 cell biological functions. Our data demonstrate the R578C mutations are severely TGOF secondary to perturbations of the various aspects of JIP3-mediated biological functions. Furthermore, our work shows that an allele-selective RNase H1–activating ASO may be an ideal therapeutic approach to treat patients with TGOF mutations in JIP3. We provided evidence that MT-JIP3 (a) inhibits cell survival and growth through overactivation of the JNK signaling pathway, (b) disrupts endosome movement and maturation along microtubules, and (c) disrupts (uncouples) dopamine 1 receptor signaling. Through these mechanisms, MT-JIP3 contributes to neurodegeneration. Moreover, in this study, we also show that a non-allele-selective ASO that reduced MT-JIP3 by >80% and WT-JIP3 by >60% was well tolerated and effective in patient-derived neuronal cells, suggesting that an ASO approach would have therapeutic potential and is expected to be an ideal approach, as it would only act on the MT mRNA.

JNK signaling is a key activator of the c-Jun transcription complex, and JNK3 has been implicated in stress responses and apoptosis. Apoptosis signal-regulated kinase 1 (ASK1) is a MAPK that activates the JNK and p38 MAPK cascades in response to environmental stresses, such as reactive oxygen species. JIP3 serves as a scaffolding protein that binds to ASK1 and enhances JNK activity induced by ASK1 and H_2_O_2_ (34). LPS is recognized by TLR4 and activates NF-κB and a group of MAPKs (35, 36). JIP3 is reported to bind TLR4 protein and participate in JNK activation of TLR downstream signaling (35). In experiments employing HeLa cells as a model, we demonstrated that overexpression of either WT-JIP3 or MT-JIP3 activated JNK signaling and inhibited cell growth. Interestingly, when the expression of JIP4 was reduced, the ability of WT-JIP3 to activate JNK signaling was significantly weakened, but the ability of MT-JIP3 to activate JNK signaling and inhibit cell growth was not changed, suggesting that the potential compensation function of JIP4 on JIP3. JIP4 and JIP3 proteins have overlapping functions in promoting axonal transport of lysosomes (11). Therefore, the high expression of JIP4 in HeLa cells alleviates the toxic effects of MT-JIP3 to a certain extent. When JIP4 expression was reduced, the toxic effect of WT-JIP3 decreased, while the toxic effect of MT-JIP3 remained unchanged (Figure 1, A and B). One likely explanation for this observation is the interaction between JIP3 and JIP4 proteins. The interaction between MT-JIP3 and JIP4 was significantly lower than that between WT-JIP3 and JIP4, which further illustrates the toxic function of MT-JIP3 that is gained. This is in line with data in other cell models with low JIP4 expression, such as patient fibroblasts and iPSC-derived neurons, where MT-JIP3 enhanced JNK signaling and reduced cell survival and growth (Figure 2, A and B, and Figure 3, B and C).

ASOs must enter the cell and reach the target RNA in the cytoplasm and nucleus to elicit antisense activity (37). PS-ASOs in EEs display Brownian-type motion and migrate only a short distance, whereas LE ASOs move linearly along microtubules over considerable distances (20, 38). Our results show that MT-JIP3 significantly interferes with ASO endosome movement in the cell body and mTOC convergence in multiple cell models. At the same time, intracellular endosome movement and mTOC convergence of multiple cargoes such as transferrin and TLR4 are delayed, which indicates that MT-JIP3 has a negative impact on intracellular transport. It is important to note that we did not observe a lysosomal defect (Figure 2F and Figure 3G), as opposed to previous observation that loss of JIP3 (*MAPK8IP3*) gene results in lysosome-filled axonal swellings and the accumulation of axonal lysosomes (4). However, in the previous study, a JIP3-depletion cell model was used to assess the function of JIP3 on lysosomes, which represents a loss of function. However, we did not observe the effect of the MT-JIP3 on lysosomes in patient-derived cells, indicating that this mutation is a gain of function, acting in the opposite direction of a loss of function previously studied (4). More importantly, neurons require active transport of proteins and organelles between the neuron cell body and axon terminals to maintain connections (39, 40). JIP3 is required for the transport of two cargos (kinases and lysosomes) from the axon terminal to the cell body (retrograde transport) (1). In multiple studies, JIP3 is involved in anterograde axonal transport through its interaction with the kinesin light chain and kinesin heavy chain components of the kinesin-1 motor (1, 7, 41). In the absence of JIP3 or JIP3 dysfunction, these cargoes accumulate, and axonal terminals become malformed, although retrograde transport of other cargoes is normal (39). Abnormalities in the “axonal” transport of MT-JIP3 may be related to the patient’s neurodevelopment disorder. Abnormal trafficking of MT-JIP3 leads to abnormal accumulation of varicosities on neuronal axons. The result of this abnormal transportation and aggregation is to increase the risk of death of nerve cells and decrease the viability of neuronal cells, thus causing neurodevelopment disorder.

The most significant differentially expressed gene found in RNA-Seq examination of iPSCs and cortical neural stem cell lines from patients with BD was the NLRP2 inflammasome (23). Pathway analysis showed that the enrichment of the *NLRP2* gene was related to the loss of dopamine receptor signaling pathway (23). We found significantly higher *NLRP2* expression in patient iPSC–derived neurons (Supplemental Figure 4A) compared with CRISPR control cells, which suggests that the patient’s dopamine receptor signaling pathway may be defective in the nervous system. The experimental results demonstrated that both patient cell models clearly showed that the presence of MT-JIP3 affected the activation of D1. The loss of brain dopamine in Parkinson’s disease is accompanied by a corresponding loss of dopamine transporters and an increase in D1 and D2 receptor density (42). This was found in brain putamen and caudate tissue in drug-naive patients and may account for the favorable early clinical response to levodopa (43). The high expression of D1 and D2 in the patient’s nerve cells further confirmed this. In addition, positron emission tomography data and in vitro data are generally consistent (43). However, long-term levodopa treatment returns receptor density to normal levels. This makes levodopa therapy a possible treatment modality (44). Nonetheless, we provide evidence that MT-JIP3 causes abnormalities in D1 activation and that abnormalities in the DA signaling and its structural receptors in the basal ganglia are the basis of Parkinson’s disease. Additionally, of course, DA is also involved in other neurodegenerative diseases, such as Huntington’s disease and multiple sclerosis (45). Changes in DA transport and DA receptor expression and signaling pathways ultimately contribute to the development of neurotoxicity, inflammation, and neurodegenerative diseases (45, 46). The dopaminergic component in this mutation also suggests that clinical assessment via dopamine scans is possible for these patients and that dysfunctional dopamine signaling may indeed contribute to their phenotype. These are in line and further support that MT-JIP3 is a severe TGOF mutation.

ASOs are typically 16- to 20-nucleotide long single-stranded oligonucleotides comprising chemically modified and natural nucleobases with modifications in the phosphate internucleotide linkage. The most commonly used phosphate replacement is PS. The PS modification increases nuclease resistance and, importantly, increases protein binding and protein binding is critical to all phases of ASO pharmacological effects (19). ASOs hybridize to specific sequences in target RNAs and can be designed to take advantage of various post- RNA binding mechanisms. ASOs can be designed to alter the function or metabolism of target RNAs by binding to sequences critical for RNA processing, stability, or translation, or they can be designed to cause degradation of target RNAs and, as a consequence, reduction in the target protein. To take advantage of this mechanism, ASOs usually incorporate high-affinity, nuclease-stable 2′ modified nucleosides in the 5′ and 3′ “wings” and deoxynucleotides in the center “gap” (47–49). From a therapeutic perspective, TGOF mutations in essential genes, pose the unique challenge of reducing the mutant RNA and protein while sparing the WT RNA and protein. To achieve this selectivity, the most common approach is to identify nonpathogenic single nucleotide variants and design ASOs to those variants. In addition to Sanger sequencing, this typically requires long-read sequencing and phasing. Since nonpathogenic single nucleotide variants and pathogenic mutations are usually independent genetic events, often several different ASOs are required to treat an entire patient population with TGOFs. Since the therapeutic objective is to reduce the protein with a TGOF, such an approach would not be useful to treat patients with loss-of-function mutations. To assess the effect of reducing JIP3 in the patient’s cells and to determine the level of selectivity for the MT-RNA and protein required, several non-allele-selective ASOs effectively inhibited the JNK signaling pathway enhanced by MT-JIP3, alleviated the abnormal cargo-laden endosomal movement caused by MT-JIP3, and thereby reduced the cell death caused by MT-JIP3 (Figure 6, E and G; Figure 7F; and Supplemental Figure 5). Importantly, the non-allele-selective ASOs demonstrated that as much as a 70% reduction of WT-JIP3 was reasonably well tolerated (Figure 6, A and C, and Figure 7, A and E), demonstrating that only modest MT selectivity is required to treat such patients. Since heterozygous TGOF mutations in essential genes are common, ASO technology is of unique value because the achievement of 10- to 20-fold MT selectivity can typically be achieved. An excellent recent demonstration of the potential of this approach is the benefit reported for a patient with a heterozygous TGOF mutation in KIF1A (50).

In conclusion, this study reveals for the first time to our knowledge that MT-JIP3 R578C causes abnormal enhancement of the JNK signaling pathway, deficient cargo endosome movement transport, and disrupted dopamine receptor responses. These abnormalities eventually lead to cell death, demonstrating that the mutation acquire a severely TGOF. It also further explains how this mutation leads to neurodegenerative diseases. Finally, this study also provides a reliable and effective ASO treatment method, which can significantly inform a subsequent therapeutic avenue and clinical treatment.

## Methods

### Sex as a biological variable.

Sex was not considered as a biological variable.

### Reagents and materials.

CellLight virus for GFP-Tubulin (C10613), GFP-Early endosome (C10586), GFP-Late endosome (C10588), CyQUANT MTT Cell Viability Assay (13154), and Pierce Protein A/G Magnetic Beads (88802) were purchased from Thermo Fisher. pcDNA3-TLR4-YFP expression plasmid (13018) was purchased from Addgene. Cy3 ChromPure Human Transferrin (009-160-050) was purchased from Jackson Immune Research Labs. LPS (L4391-1MG) was from MilliporeSigma.

siRNAs and primer probe sets are listed in Supplemental Table 1; antibodies are listed in Supplemental Table 2; and ASOs are listed in Supplemental Table 3.

### Cell culture.

HeLa cells, unaffected human skin fibroblasts, and patient skin fibroblasts were grown in DMEM with 10% FBS, 0.1 μg/mL streptomycin, and 100 units/mL penicillin. HeLa cells were seeded at 70% confluency, 1 day before siRNA transfection. siRNAs were transfected using lipofectamine RNAiMAX (Invitrogen, 13778075) according to the manufacturer’s protocol at 7.5 nM final concentration unless contraindicated. 24 hours after siRNA transfection, cells were again transfected with 1 μg/mL of expression plasmids using lipofectamine 2000 (Invitrogen, 11668019), according to manufacturer’s protocol. 24 hours after plasmids transfection, cells were reseeded into 96-well plates for cell proliferation or PS-ASO activity or uptake assays or were seeded into 35 mm poly-D-lysine–coated glass-bottom dishes (MatTek, P35GC-0-14-C) at 70% confluency for immunofluorescence staining and live imaging.

Three human dermal control fibroblasts (GM02936, GM03529 and GM03440) were purchased from Coriell Institute for Medical Research and cultured in DMEM with 10% FBS.

The patient fibroblasts were isolated from a skin biopsy, and an iPSC line was generated from the PBMCs of a 2.5-year-old boy with global developmental delay, who was diagnosed as having a MAPK8IP3 NM_015133.5 (MAPK8IP3):c.1732C>T (p.Arg578Cys) (R578C) missense mutation. Patient cell line generation was carried out using the standard method as described in the referenced protocol (51).

### CRISPR control iPSCs.

iPSCs from a patient carrying the MAPK8IP3 c.1732C>T (p.Arg578Cys) variant were generated by the Columbia Stem Cell Core facility (Columbia University, New York, New York, USA) according to (51). The CRISPR-Cas9 corrected lines were generated by the Columbia Stem Cell Core facility according to (52). Validated synthetic gRNAs were designed using the CRISPR Design Tool (https://design.synthego.com/#/, Synthego) to target the following variant: NM_015133.5 (MAPK8IP3); Chr16, position 1762843 (GRCh38) MAPK8IP3 c.1732C>T, AA change p. Arg578Cys.

To target the variant in MAPK8IP3, the commercially synthetized (Synthego) sgRNA GCTGAAGCTGCCAGAGGCACPAMAGG was used in combination with the single-stranded oligodeoxynucleotide (Integrated DNA Technologies): AGGGAGGTTCCCTGGTCCTCTGCCCACCCCTCACCTCTCTGTGCCTCTGGCAGCTTCAGCCGCCTCTTCAGCTCTTCCTCCAGCCCCCCTCCGGCCAA. The PAM was silenced by altering C>T, with the target editing T>C.

The gRNAs were chosen based on their cutting efficiency and better off-target score. The single-stranded oligodeoxynucleotides were designed as a complementary sequence to the sgRNA target strands. 15 μg gRNAs and 15 μg donor DNA were incubated for 25 minutes at room temperature in combination with 10 μg Alt-R S.p. HiFi Cas9 Nuclease V3 (Integrated DNA Technologies) to generate the RNP complex that was mixed with 1.0 × 10^6^ of the target iPSCs. Electroporation was performed using the 4D- Nucleofector (Lonza, program CA137). The Inference of CRISPR Edits online tool (Synthego) was used to determine cleavage and knockin efficiency. 48–72 hours after electroporation cells were seeded at low density (single cell) and then colonies were manually picked for genotyping. Correction of the target sequence was confirmed with Sanger sequencing. Primer set forward GCCAGAGAGTCGCAGGTAAG and reverse CAGGGAAGAATTCCAGGGGC were used to amplify the target region. Sanger sequencing was performed to confirm editing using the sequencing primer GAAGTCGACCATCTGCAG.

### iPSC-derived neuronal cells.

For routine maintenance, iPSCs were cultured on Geltrex-coated (Thermo Fisher, A14133-02) plates in mTeSR Plus medium (Stemcell Technologies, 100-0276) supplemented with 1× Penicillin-Streptomycin (Thermo Fisher, 15140122). When the cultures reached 70%–90% confluence, the cells were passaged as aggregates using Versene solution (Thermo Fisher, 15040066) and replated into mTeSR Plus medium supplemented with 1× Penicillin-Streptomycin and 10 μM Y-27632 (SelleckChem, S1049). Quality of the iPSC lines was monitored daily to ensure they remained free from spontaneous differentiation. All iPSC lines were maintained under these routine culture conditions for at least 2 weeks before initiating neuronal differentiation.

Four days before the start of differentiation, iPSCs were passaged and seeded at an appropriate density to yield 55%–70% confluence by the start of differentiation. Neuronal differentiation was performed using Quick-Neuron EX-SeV (Elixirgen Scientific) according to the manufacturer’s protocol, with the following minor modifications. On day 0, cells were passaged using diluted Accutase (1:1 dilution in DPBS) (Stemcell Technologies, 07920), transduced with viruses as per the manufacturer’s protocol, and were plated onto Geltrex-coated (Thermo Fisher, A14133-02) 6-well plates. On day 3, cells were passaged and were plated onto 96-well, 6-well, or 8-well chamber slides coated with Poly-L-ornithine (Sigma, P4957)/Laminin (Thermo Fisher, 23017015) at 135,000 cells/cm^2^ for various assays. Starting from day 10, the cells were maintained in Neurobasal plus medium (Thermo Fisher, A3582901) supplemented with B27 plus, with partial media changes performed twice weekly. Cells were used for various assays from day 14 onward.

### cAMP assay.

Cells were cultured in 96-well plates (10,000 cells/well) and treated with ligands for different time points after overnight postplating. Cells were washed gently with PBS and cAMP assay was performed using Promega cAMP-Glo Assay Kits (V1502, Promega). 25 μL lysis buffer was added to each well and incubated at room temperature for 10 minutes. 5 μL the lysate was used to conduct BCA analysis using Pierce BCA Protein Assay Kit. The rest of the 20 μL of lysate was used to measure cAMP according to the manufacturer’s protocol (cAMP-Glo Assay, Promega). cAMP levels were normalized to absolute protein levels for each well.

### Cell proliferation and cell viability.

Cell proliferation was measured using MTT (3-(4,5-dimethylthiazol-2-yl)-2,5-diphenyl-2H-tetrazolium bromide) assay (Thermo Fisher, V13154). Cells were seeded at 2,000 cells/well (HeLa) or 3,000 cells/well (fibroblasts) in 96-well plates and cultured in DMEM medium with 10% FBS after 48 hours or 72 hours ASO transfection. Cells were incubated in 10% MTT substrate in DMEM for 4 hours at 37ºC and then washed gently with 1× PBS. Residual precipitate was then dissolved in 50 μL DMSO per well. The optical density of converted substrate was measured at 570 nm, and the relative MTT was presented as a percentage of untreated control groups with cells treated with ASOs.

Cell viability was measured by trypan blue cell counting assay (Fisher, 15-250-061). The treatment of cells was the same as above. After treatment, cells were harvested using 0.25% Trypsin-EDTA (Thermo Fisher, 25200056), stained by 0.4% trypan blue (Thermo Fisher,15250061), and then counted by Countess 3 (Thermo Fisher, AMQAX2000). Both cell number of viable and death cells were recorded, and the relative cell viability was presented as percent of untreated control groups with cells treated with ASOs.

### Western blot and immunoprecipitation assay.

Cells were treated with ASOs for 48 hours. Cell lysates were processed and run through SDS-PAGE gel electrophoresis. An anti-GAPDH antibody was obtained from Santa Cruz Biotechnology and was used in 1:8,000 dilution (sc-47724). All other antibodies were obtained from Santa Cruz and were used in a 1:1,000 dilution: D1DR (sc-33660), D2DR (sc-5303), JIP3 (sc-46663), and JIP4 (sc-271492). Phospho-AKT S473 (no. 9271), total AKT (no. 9272), Phospho-JNK (Thr183/Tyr185) (no. 9251), total JNK (no. 9252), and p-c-Jun (ser 73) (no. 3270) antibodies were obtained from Cell Signaling. JIP3 (25212-1-AP) antibody was purchased from ProteinTech for Western blot specifically. For each experiment, protein lysates were loaded into multiple gels with 30 μg total proteins per lane and processed simultaneously to separately probe for antibodies specific to phosphorylated proteins and total proteins. All membranes were probed with GAPDH as loading controls, and a representative loading control was used in the figures. Western blotting images were developed by enhanced chemiluminescent substrate (Cytiva, RPN223) and imaged on Bio-Rad Chemidoc imaging system, and figures present representative results from 1 experiment of at least 3 independent experiments.

For IP assay, cell lysates were incubated with 7 μg antibodies (Supplemental Table 2) overnight at 4ºC and then the protein/antibody mixture was incubated with 35 μL Pierce protein A/G magnetic beads overnight at 4ºC. After incubation, the beads were collected with a magnetic stand and washed with wash buffer (50 mM Tris pH 7.4 and 150 mM NaCl) 5 times; then, samples were eluted by 100 μL 1× NuPage LDS loading buffer (with 5% 2-mercaptoethanol). Samples were boiled for 5 minutes, 20 μL of each gel was loaded for Western blot, and 20% inputs were applied.

### RNA preparation and RT-qPCR.

Cells cultured in 96-well plates were lysed using 40 μL Guanidine Isothiocyanate Solution (Thermo Fisher, 15577018) followed by 1:1 addition of 70% ethanol. The lysates were passed through 384-well PALL plates (VWR, 16003-810) using a vacuum and washed with 45 μL RNA wash buffer (60% ethanol, 60 mM potassium acetate, 10 mM Tris-HCl pH 7.5) before being incubated with 1 unit of DNAse (Invitrogen, 18-047-019) for 20 minutes. The columns were then washed twice with 40 μL 1:4 Guanidine Isothiocyanate and twice with 60 μL RNA wash buffer. RNA was eluted in 50 μL ultrapure water. RT-qPCRs were performed using AgPath-ID One-Step RT-PCR Reagents (4387391) according to the manufacture’s protocol. mRNA levels were normalized to the amount of total RNA present in each reaction as determined for duplicate RNA samples using the Quant-it RiboGreen RNA Assay Kit (Life Technologies, R11491) according to the manufacturer’s instructions.

### Immunofluorescence staining and confocal imaging.

Cells cultured in DMEM containing 10% FBS were fixed with 4% paraformaldehyde in PBS 15 minutes at room temperature, permeabilized with 0.3% Triton X-100 for 5 minutes, and blocked with 4% goat serum and 1% BSA for 1 hour. Fixed cells were incubated with primary antibodies (1:100 to 1:250) (Supplemental Table 2) in the blocking buffer overnight. After washing 3 times with PBST (0.1% Tween 20 in PBS), cells were incubated for 1 hour with secondary antibody conjugated with fluorophores (1:1,000). After washing 3 times with PBST, cells were mounted with anti-fade reagent containing DAPI (Life Technologies). Cells were imaged using a Keyence fluorescence microscope. Analysis of images was carried out using Fiji ImageJ (NIH) and MS PowerPoint.

Live-cell imaging was performed by culturing cells in glass-bottom dishes, followed by 24 hour virus transduction, and expressing various GFP-labeled markers for tubulin, EE, or LE. Next, cells were incubated with 1 μM Cy3-transferrin (or Cy3-ASOs for HeLa cells) for different time periods, as indicated in figure legends, with or without treatment by ASOs. Medium was then changed to prewarmed Opti-MEM reduced serum medium (Life Technologies, 31985070), and images were continuously recorded using a ×63 objective lens and 2 μs/pixel sampling speed in an environmental chamber at 37ºC for 2–10 minutes. Videos and images were generated using LAS X microscope software (Leica). Time projection data were generated from 2-minute videos, by overlaying all continuous images taken at approximately 3-second intervals.

### Quantification and measurement of images.

Measurement of the movement distances and speed of ASO foci was performed using Imaris (Oxford Instruments) (20). Time-lapse videos were generated using the “spots” function, which included automatic background subtraction and an autoregressive motion algorithm for particle tracking. For each cell, the mean speed and total PS-ASO trafficking distance were measured every 1.25 seconds over a period of approximately 60 seconds, focusing on the cell periphery, perinuclear area, and the entire cell. The mean speeds and total distances for each time point were plotted in Excel, based on data from about 10 cells. *P* values were calculated using an unpaired *t* test in Prism.

### STORM imaging and Nanoimager microscopy.

Sample preparation for STORM followed the well-established protocol reported in ref. 53. Briefly, cultured CRISPR control and patent iPSC–derived neurons were fixed with 4% PFA in PBS for 2 minutes, subsequently washed 3 times with PBS, and then permeabilized with 0.15% (vol/vol) Triton X-100 in PBS for 5 minutes. Samples were blocked with 3% BSA in PBS for 1 hour at room temperature and then incubated with primary antibodies (Supplemental Table 2) overnight at 4ºC. After overnight incubation, samples were washed 3 times with PBS and then stained with Alexa dye conjugated secondary antibodies (Supplemental Table 2) with Alexa Fluor 647 and Alexa Fluor 488 for 1 hour at room temperature. After washed 3 times in PBS, samples were ready for STORM. The high-resolution experiment was performed using a Nanoimager S Mark III microscope from ONI. STORM images of JIP3, Rab7, and KIF5b antibodies (Supplemental Table 2) of CRISPR control and patient cells were obtained using a ONI Nanoimager equipped with ×100 oil immersion objective with 1.5 NA in TIRF mode and s-CMOS camera. The images were processed using NimOS software. All STORM images were subject to automatic drift correction and filtered for a localization precision of 30 nm in *x* and *y* coordinates through NimOS software after acquisition. Data were then exported into csv files and converted into bin files.

### Statistics.

For in vitro assays, all quantitative data were reproduced in at least 3 independent biological experiments, with multiple replicates per experiment. Between-group comparisons were performed with a 2-tailed Student’s *t* test, and data were expressed as mean ± SD. The cutoff criteria for significance across the manuscript was *P* < 0.05. Statistical analyses were performed from 3 independent biological experiments using Prism with unpaired 2-tailed *t* test for curve comparison based on nonlinear regression (dose-response curves) for *xy* analyses, using the equation log (agonist) versus normalized response – variable slope. The *y* axis (relative level) was used as the normalized response.

### Study approval.

This study was approved by the Human Research Protection Office, Institutional Review Board, Columbia University.

### Data availability.

Values for all data points in graphs are reported in the Supporting Data Values file.

## Author contributions

WZ, KSS, and STC designed the study and wrote the paper. WZ performed the experiments. SM performed Western blot experiments and cAMP measurement. RT and AF performed the iPSC differentiation to neuronal cells. WKC performed the patient biopsy collection and patient iPSC generation. RNB performed STORM. All authors analyzed the data.

## Supplementary Material

Supplemental data

Supplemental video 1

Supplemental video 2

Supplemental video 3

Supplemental video 4

Supporting data values

## Version 1 03/20/2025

In-Press Preview

## Version 2 04/22/2025

Electronic publication
