# Supplementary material for: A toxic gain-of-function variant in MAPK8IP3 provides insights into JIP3 cellular roles
Source: JCI Insight. 2025 Mar 20;10(8):e187199. doi: 10.1172/jci.insight.187199 (PMC12016931; doi:10.1172/jci.insight.187199)
Supplement: Supplemental data [file jciinsight-10-187199-s256.pdf]

Supplementary Figure Legends

Supplementary Figure 1. Hela cells were transfected with siRNAs (siLuc or siJIP4) for overnight and recovered in fresh culture medium for 24 hours, and then transfected with expression plasmids encoding either Flag tagged WT-JIP3 or MT-JIP3 for overnight following 24 hours recovery in fresh culture medium for further experiments. (A) the levels of JIP3, Flag-JIP3 and JIP4 proteins were measured by western blot for validation of transfection. (B) Cell proliferation was measured by MTT assay. Cells transfected with different concentrations of siJIP4. siCtrl was used as controls to normalize the data. n = 3, * P < 0.05, ** P < 0.01. (C) The quantification of western blots for co-IP in Fig.1D. (D) The quantification of the colocalization (120 mins) between Rab7 and Cy3-ASO in Fig. 1E was calculated by Image J using Person's R value. n= 15, ** P < 0.01. (E) The quantification of the Cy3 integrated fluorescence density in the mTOC area (120 mins) in Fig. 1E was calculated by Image J. n= 10, ** P < 0.01. (F) Videos were recorded for different siRNA/plasmid treated HeLa cells were incubated with 1 μM Cy3-labeled PS-ASO 446654 for different time points. The colocalization of Cy3-ASOs and Rab7 (Late endosome marker, LE) were observed by Keyence X800 microscope.

Supplementary Figure 2. (A) The quantification of the colocalization between Rab7 and Cy3-transferrin (120 mins) in Fig. 2D was calculated by Image J using Person's R value. (B) The quantification of the colocalization between Rab5/7 and Cy3-transferrin in Fig. 2E was calculated by Image J using Person's R value. The cell imaging of either patient fibroblasts or healthy control fibroblasts were co-stained with JIP3 and Rab5 (early endosome marker) (C), and JIP3 and Rab7 (late endosome marker) (D). The

colocalization was observed by Keyence BZ-X800 microscope. Colocalization was quantified with Pearson’s coefficient by ImageJ. (E) The interaction of JIP3 and JIP4 in control and patient fibroblasts was detected by co-IP. Briefly, Flag antibody was used for pulling down cell lysates and JIP3/4 proteins were measured by western blot.

Supplementary Figure 3. (A) The quantification of number of varicosities per sample was analyzed by ImageJ through the function of “analyze particle”. Varicosities were represented as particle clusters. ** P< 0.01. (B) Crispr control and patient iPSC derived neurons were fixed and stained with anti-βIII tubulin antibody. Varicosities in long axons were detected by Keyence BZ-X800 microscope with different magnifications. Representative varicosities were pointed out by white arrows. (C) The cell imaging of either CRISPR control or patient iPSC derived neurons were co-stained with JIP3 and Rab5 (early endosome marker). The colocalization was observed by Keyence BZ-X800 microscope. Colocalization was quantified with Pearson’s coefficient by ImageJ. (D) The quantification of the colocalization of Cy3-PS-ASO and Rab7 in iPSC derived neurons in Fig 3E was calculated by Image J using Person’s R value. * P < 0.05. (E) The quantification of the colocalization of JIP3 and KIF3B in iPSC derived neurons in Fig 3F was calculated by Image J using Person’s R value. * P < 0.05. (F) The interaction of JIP3 and KIF5b in CRISPR control and patient iPSC derived neuronal cells was detected by co-IP. Briefly, Flag antibody was used for pulling down cell lysates and JIP3 and KIF5b proteins were measured by western blot.

Supplementary Figure 4 (A) mRNA levels of *NLRP2* either in CRISPR control or patient iPSC derived neurons were measured by qPCR. (B) Three healthy subjects derived fibroblasts (2936, 3440 and 3529) or patient fibroblasts were seeded in 96 wells (8000 cells/well). Basal cAMP levels were measured by cAMP-Glo™ Assay (Promega). n = 3.

Supplementary Figure 5. Non-allele selective PS-ASOs rescue patient derived fibroblasts from MT-JIP3 induced cell toxicity. Three different health controls (2934, 3529 and 3440) and patient fibroblasts were treated with three different non-allele selective ASO1 (1713794), 687 ASO2 (1713687) and 510 ASO3 (1713510) targeting MAPK8IP3 (JIP3) for 3 days. (A) After 24 hours incubation, RNA was extracted by GITC assay and *MAPK8IP3* mRNA level was measured q-PCR. Data was normalized to UTC of control cells. n = 3, ** $P < 0.01$. (B) *SPAG9* (JIP4) mRNA level was measured q-PCR. Data was normalized to UTC of control cells. n = 3, ** $P < 0.01$. (C) After 72 hours incubation, cell proliferations were measured by MTT assay and normalized to UTC of control cells. n = 3, * $P < 0.05$, ** $P < 0.01$. (D) The quantification of the colocalization (2 hrs.) between Rab7 and Cy3-Transferrin in Fig. 6G was calculated by Image J using Person's R value. n= 10, ** $P < 0.01$.

Supplementary Figure 6. Non-allele selective PS-ASOs rescue patient iPSC derived neurons from MT-JIP3 induced cell death **(**A) Expression of the MT-JIP3 in patient iPSC derived neurons leads to increased risk of death compared with CRISPR Control iPSC derived neurons. A representative bright filed images of three iPSC derived neurons,

CRISPR control, Patient and Patient treated with ASO2 (5 μM) at day 42 are shown (lower-panel). Images were taken by Keyence microscope. Cell viability was measured by trypan blue cell count assay and normalized to UTC of CRISPR control cells (right panel). * $P < 0.05$.

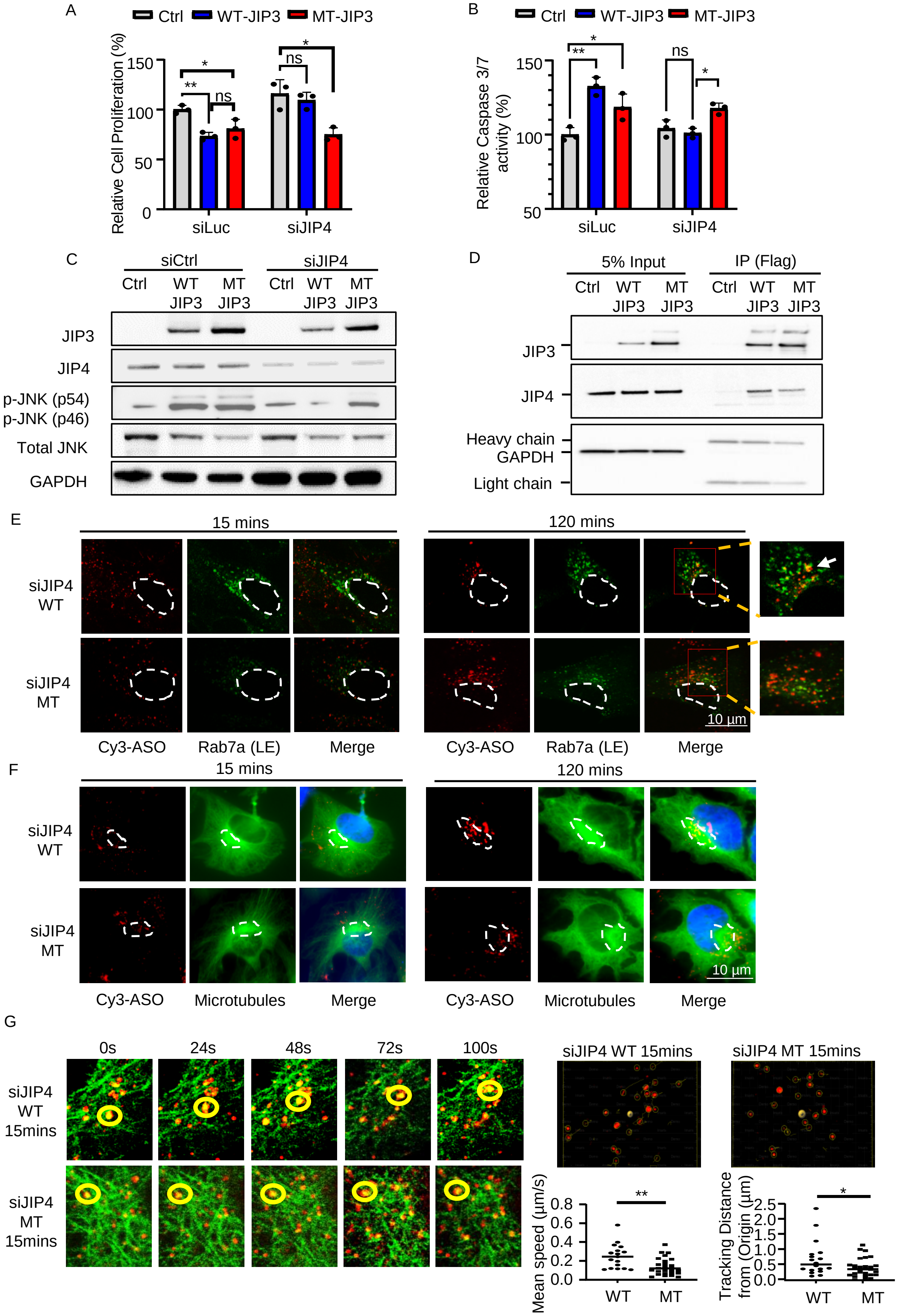

A
Ctrl
WT-JIP3
MT-JIP3
Relative Cell Proliferation (%)
siLuc
siJIP4
B
Relative Caspase 3/7 activity (%)
C
siCtrl
siJIP4
JIP3
JIP4
p-JNK (p54)
p-JNK (p46)
Total JNK
GAPDH
D
5% Input
IP (Flag)
Heavy chain
Light chain
E
15 mins
120 mins
siJIP4 WT
siJIP4 MT
Cy3-ASO
Rab7a (LE)
Merge
10 µm
F
Microtubules
G
0s
24s
48s
72s
100s
siJIP4 WT 15mins
siJIP4 MT 15mins
Mean speed (µm/s)
Tracking Distance from (Origin (µm)
WT
MT

Figure 2

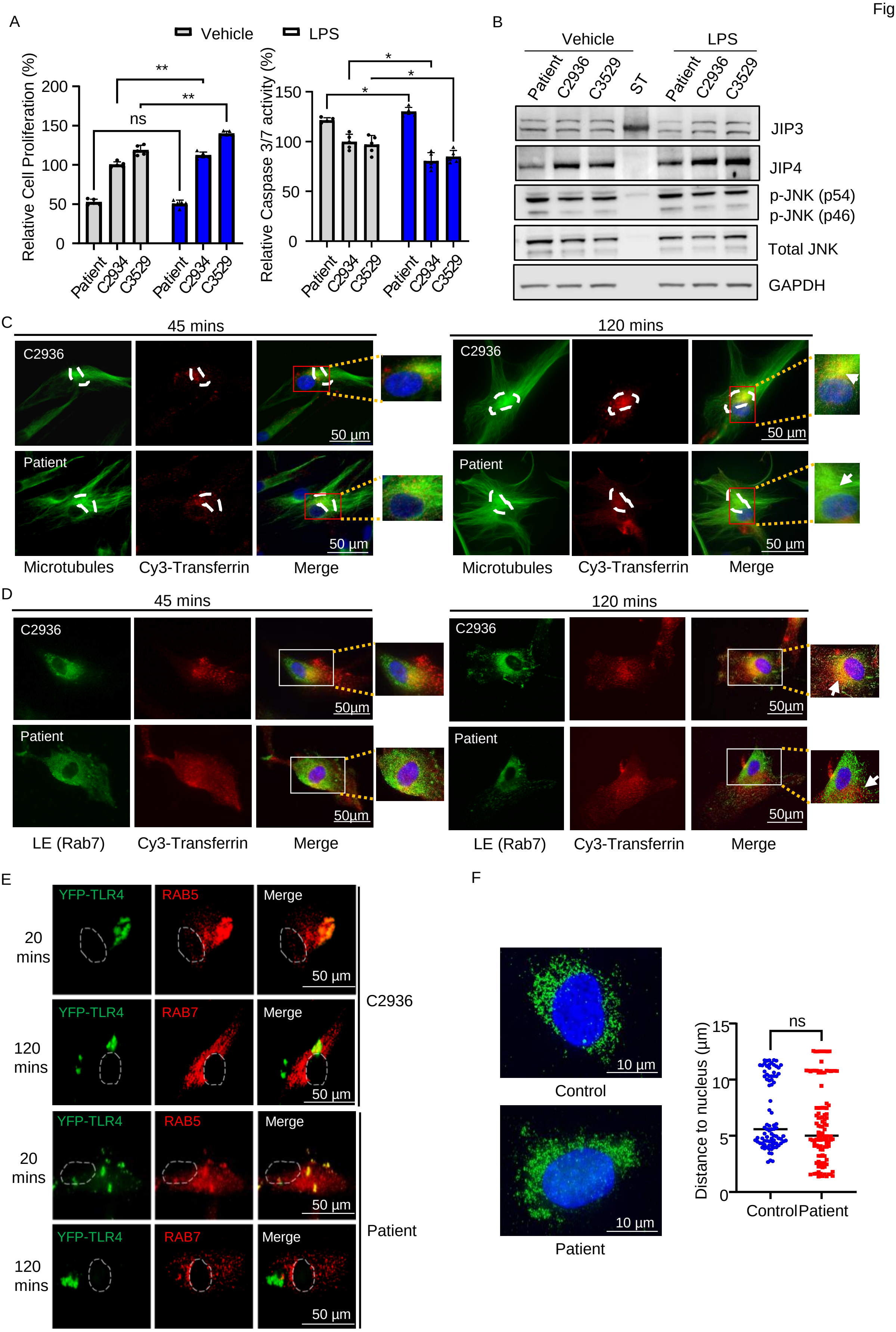
A
Vehicle
LPS
Relative Cell Proliferation (%)
**
**
ns
Patient
C2934
C3529
Relative Caspase 3/7 activity (%)
*
B
JIP3
JIP4
p-JNK (p54)
p-JNK (p46)
Total JNK
GAPDH
Patient
C2936
ST
C
45 mins
120 mins
50 µm
Microtubules
Cy3-Transferrin
Merge
D
50µm
LE (Rab7)
E
YFP-TLR4
RAB5
RAB7
20 mins
120 mins
C2936
F
10 µm
Control
Distance to nucleus (µm)

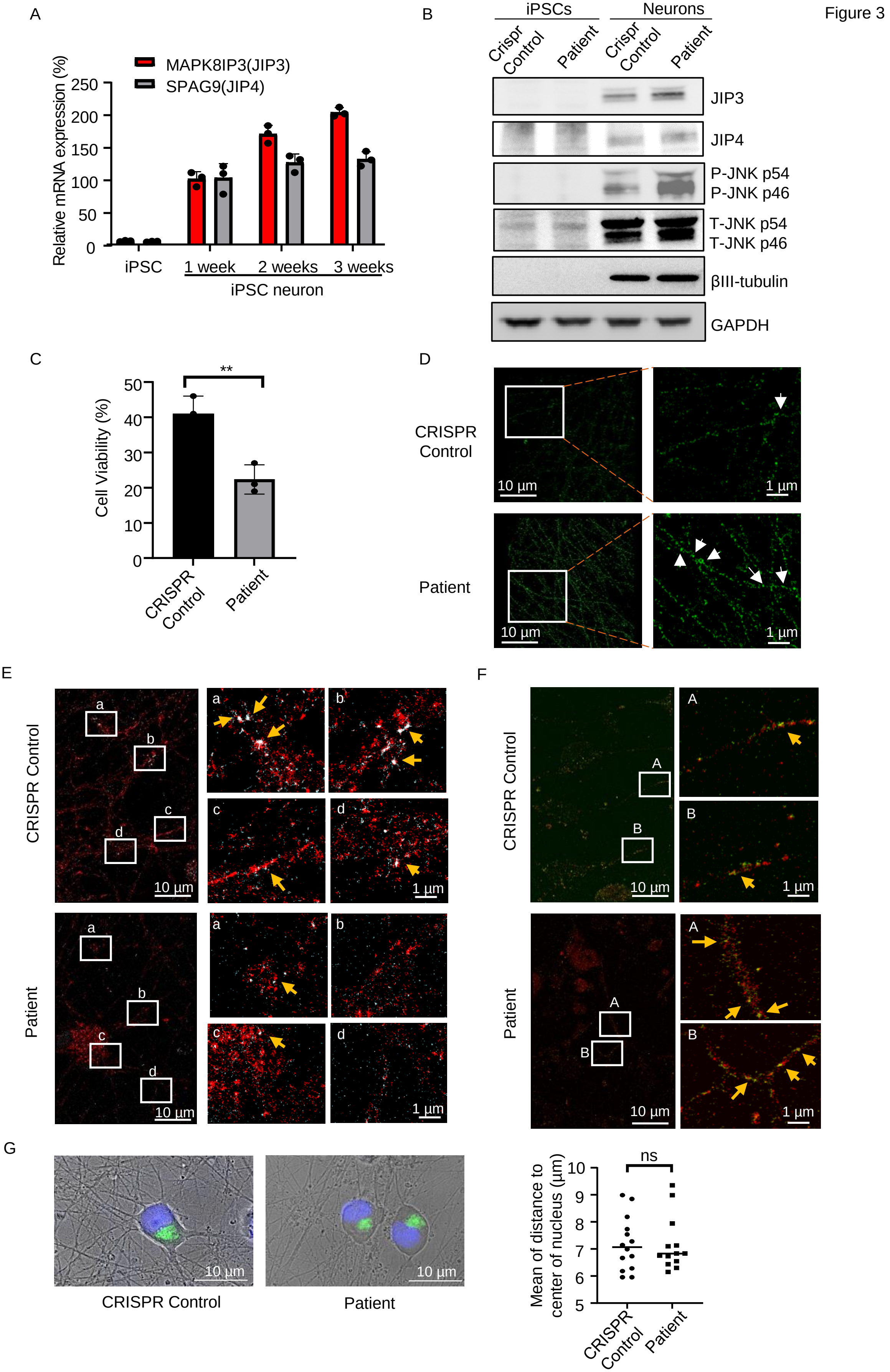

Figure 3
A
MAPK8IP3(JIP3)
SPAG9(JIP4)
Relative mRNA expression (%)
250
200
150
100
50
0
iPSC
1 week
2 weeks
3 weeks
iPSC neuron
B
iPSCs
Neurons
Crispr Control
Patient
Crispr Control
Patient
JIP3
JIP4
P-JNK p54
P-JNK p46
T-JNK p54
T-JNK p46
βIII-tubulin
GAPDH
C
**
Cell Viability (%)
50
40
30
20
10
0
CRISPR Control
Patient
D
CRISPR Control
Patient
10 µm
1 µm
E
CRISPR Control
Patient
a
b
c
d
10 µm
1 µm
F
CRISPR Control
Patient
A
B
10 µm
1 µm
G
CRISPR Control
Patient
10 µm
ns
Mean of distance to center of nucleus (µm)
10
9
8
7
6
5
CRISPR Control
Patient

Figure 4

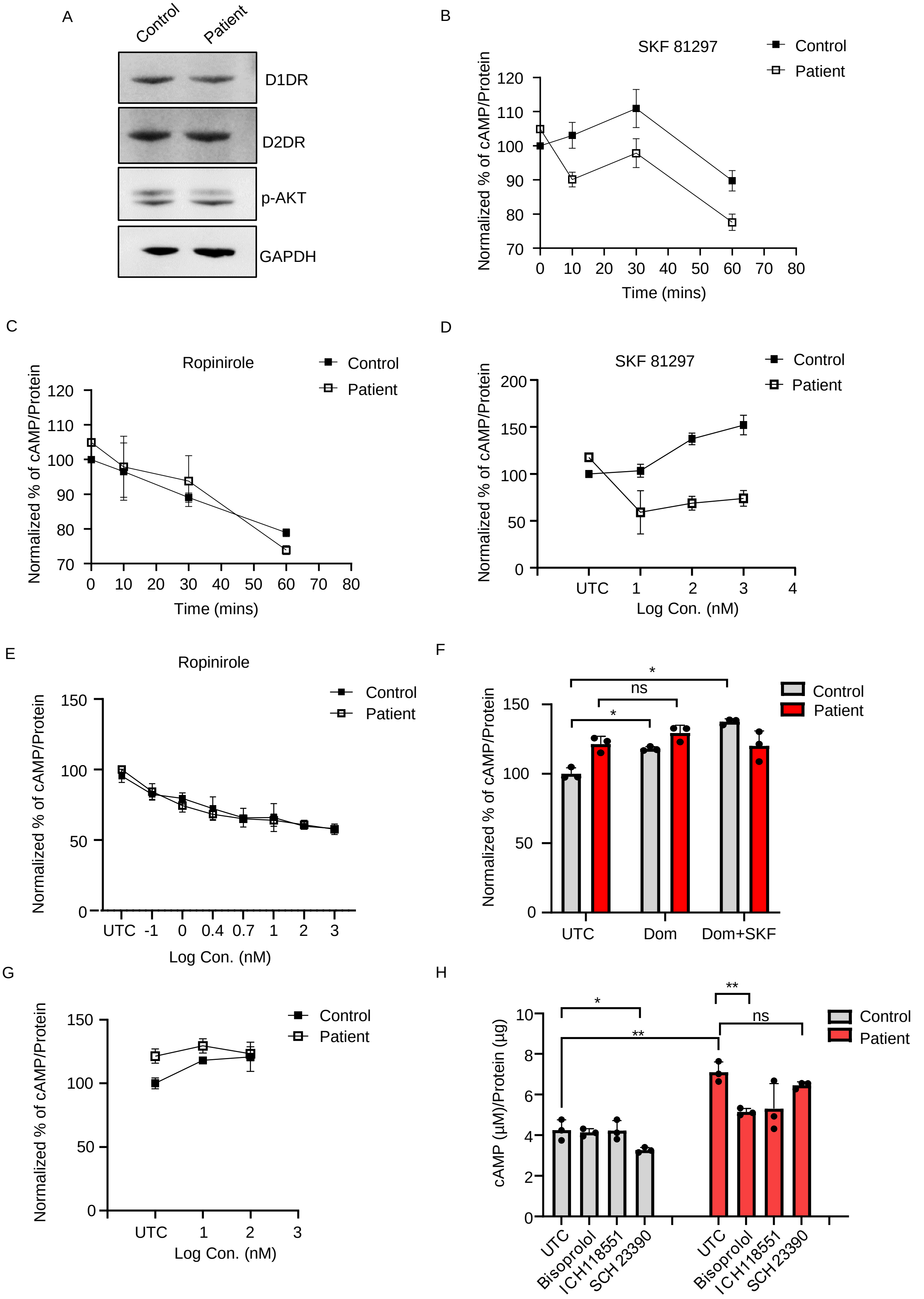

A
Control
Patient
D1DR
D2DR
p-AKT
GAPDH
B
SKF 81297
Control
Patient
Normalized % of cAMP/Protein
Time (mins)
C
Ropinirole
Control
Patient
Normalized % of cAMP/Protein
Time (mins)
D
SKF 81297
Control
Patient
Normalized % of cAMP/Protein
UTC
Log Con. (nM)
E
Ropinirole
Control
Patient
Normalized % of cAMP/Protein
UTC
Log Con. (nM)
F
Control
Patient
Normalized % of cAMP/Protein
ns
UTC
Dom
Dom+SKF
G
Control
Patient
Normalized % of cAMP/Protein
UTC
Log Con. (nM)
H
Control
Patient
cAMP (µM)/Protein (µg)
ns
UTC
Bisoprolol
ICH118551
SCH 23390

Figure 5

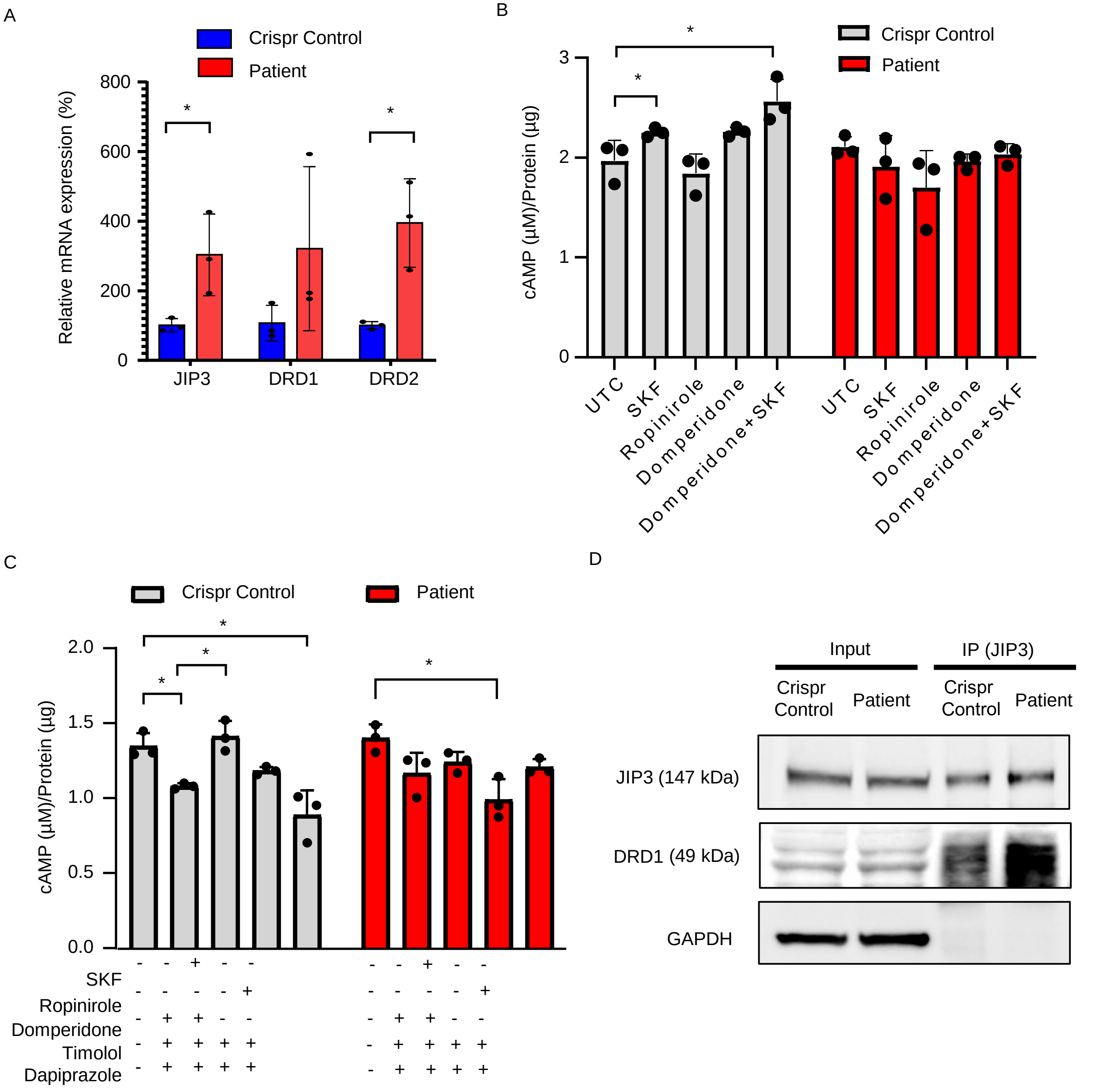
A
Crispr Control
Patient
Relative mRNA expression (%)
800
600
400
200
0
*
*
JIP3
DRD1
DRD2
B
Crispr Control
Patient
cAMP (µM)/Protein (µg)
3
2
1
0
*
*
UTC
SKF
Ropinirole
Domperidone
Domperidone+SKF
UTC
SKF
Ropinirole
Domperidone
Domperidone+SKF
C
Crispr Control
Patient
cAMP (µM)/Protein (µg)
2.0
1.5
1.0
0.5
0.0
*
*
*
*
SKF
Ropinirole
Domperidone
Timolol
Dapiprazole
D
Input
IP (JIP3)
Crispr Control
Patient
Crispr Control
Patient
JIP3 (147 kDa)
DRD1 (49 kDa)
GAPDH

Figure 6

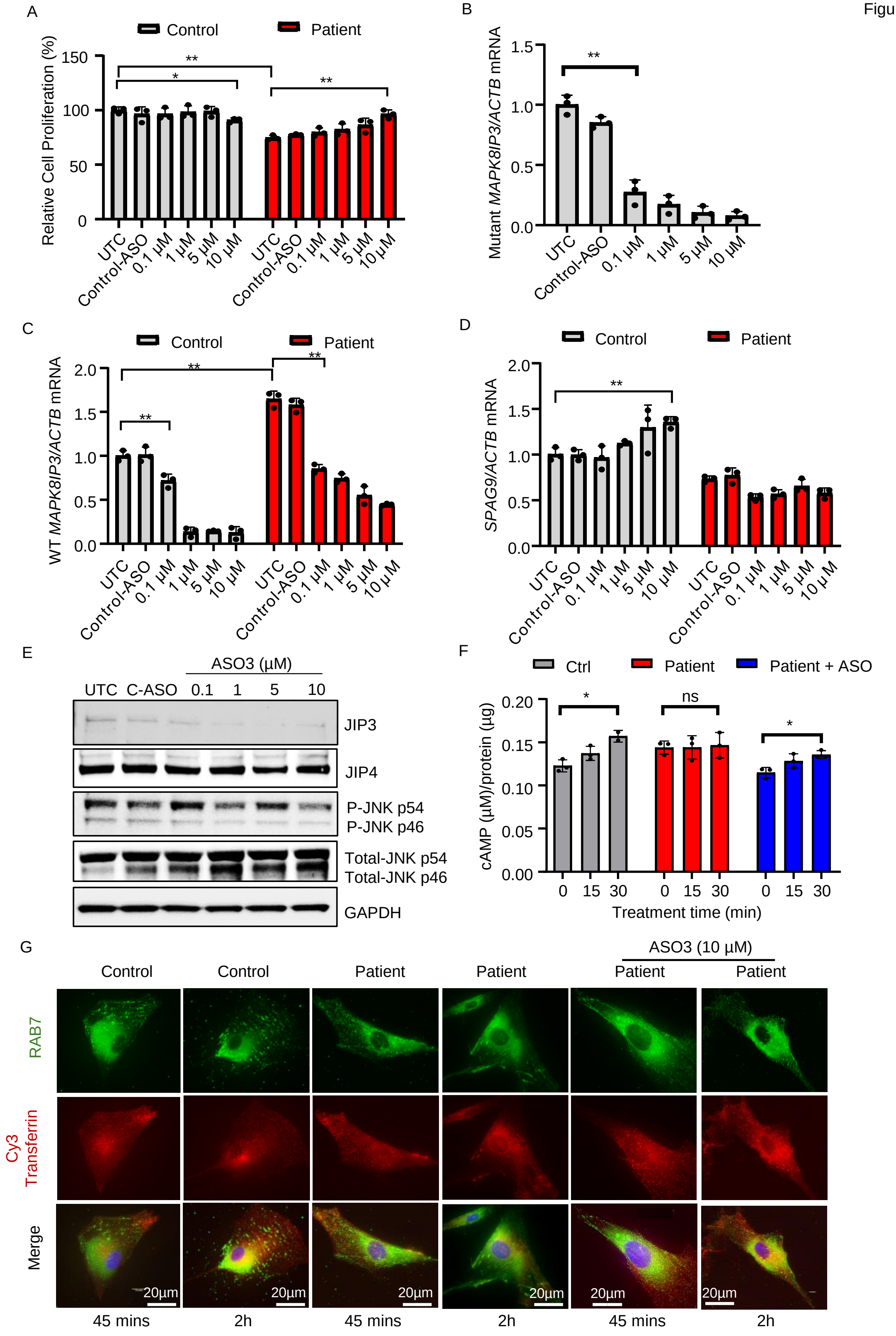
A
Control
Patient
Relative Cell Proliferation (%)
UTC
Control-ASO
0.1 μM
1 μM
5 μM
10 μM
B
Mutant MAPK8IP3/ACTB mRNA
C
WT MAPK8IP3/ACTB mRNA
D
SPAG9/ACTB mRNA
E
ASO3 (μM)
UTC C-ASO 0.1 1 5 10
JIP3
JIP4
P-JNK p54
P-JNK p46
Total-JNK p54
Total-JNK p46
GAPDH
F
Ctrl
Patient
Patient + ASO
cAMP (μM)/protein (μg)
Treatment time (min)
ns
G
ASO3 (10 μM)
Control
Patient
RAB7
Cy3 Transferrin
Merge
20μm
45 mins
2h

Figure 7

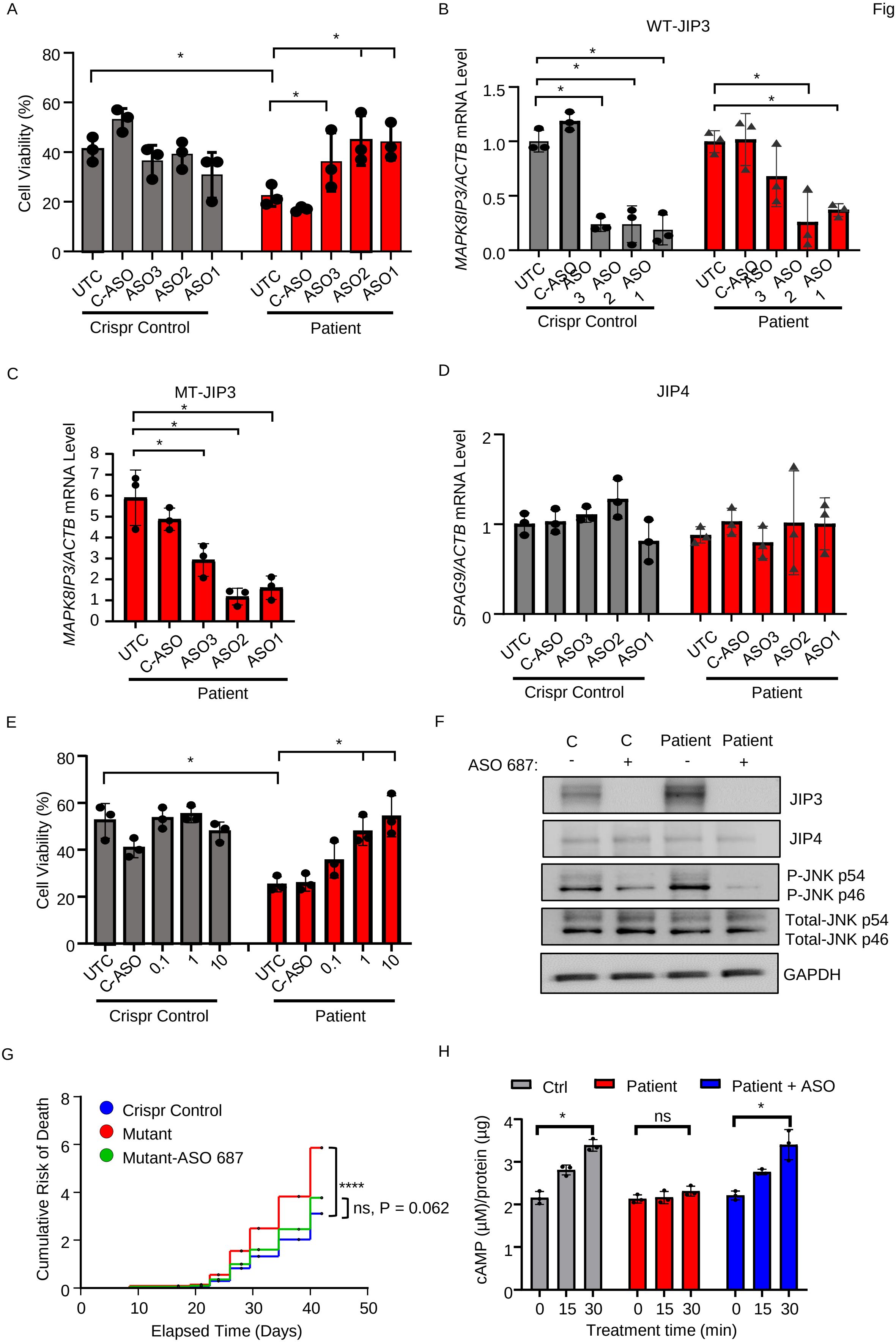
A
Cell Viability (%)
80
60
40
20
0
*
*
*
UTC
C-ASO
ASO3
ASO2
ASO1
Crispr Control
Patient
B
WT-JIP3
MAPK8IP3/ACTB mRNA Level
1.5
1.0
0.5
0.0
ASO 3
ASO 2
ASO 1
C
MT-JIP3
8
7
6
5
4
3
2
1
0
D
JIP4
SPAG9/ACTB mRNA Level
2
1
E
0.1
1
10
F
C
Patient
ASO 687:
-
+
JIP3
JIP4
P-JNK p54
P-JNK p46
Total-JNK p54
Total-JNK p46
GAPDH
G
Cumulative Risk of Death
Crispr Control
Mutant
Mutant-ASO 687
****
ns, P = 0.062
Elapsed Time (Days)
H
Ctrl
Patient
Patient + ASO
cAMP (μM)/protein (μg)
ns
Treatment time (min)

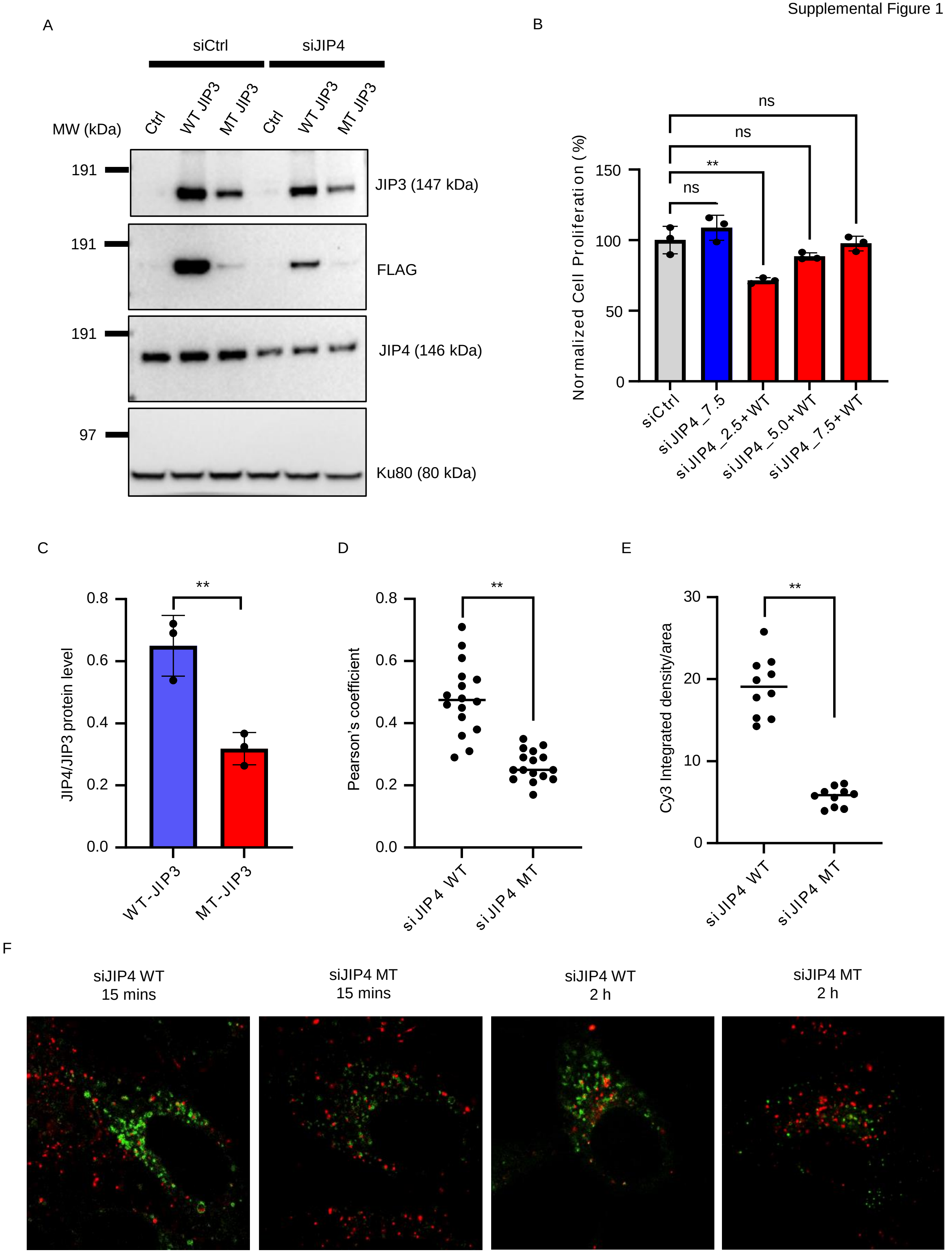
A
siCtrl
siJIP4
Ctrl
WT JIP3
MT JIP3
Ctrl
WT JIP3
MT JIP3
MW (kDa)
191
191
191
97
JIP3 (147 kDa)
FLAG
JIP4 (146 kDa)
Ku80 (80 kDa)
B
Normalized Cell Proliferation (%)
ns
ns
**
ns
siCtrl
siJIP4_7.5
siJIP4_2.5+WT
siJIP4_5.0+WT
siJIP4_7.5+WT
C
JIP4/JIP3 protein level
**
WT-JIP3
MT-JIP3
D
Pearson's coefficient
**
siJIP4 WT
siJIP4 MT
E
Cy3 Integrated density/area
**
siJIP4 WT
siJIP4 MT
F
siJIP4 WT
15 mins
siJIP4 MT
15 mins
siJIP4 WT
2 h
siJIP4 MT
2 h

Supplemental Figure 2

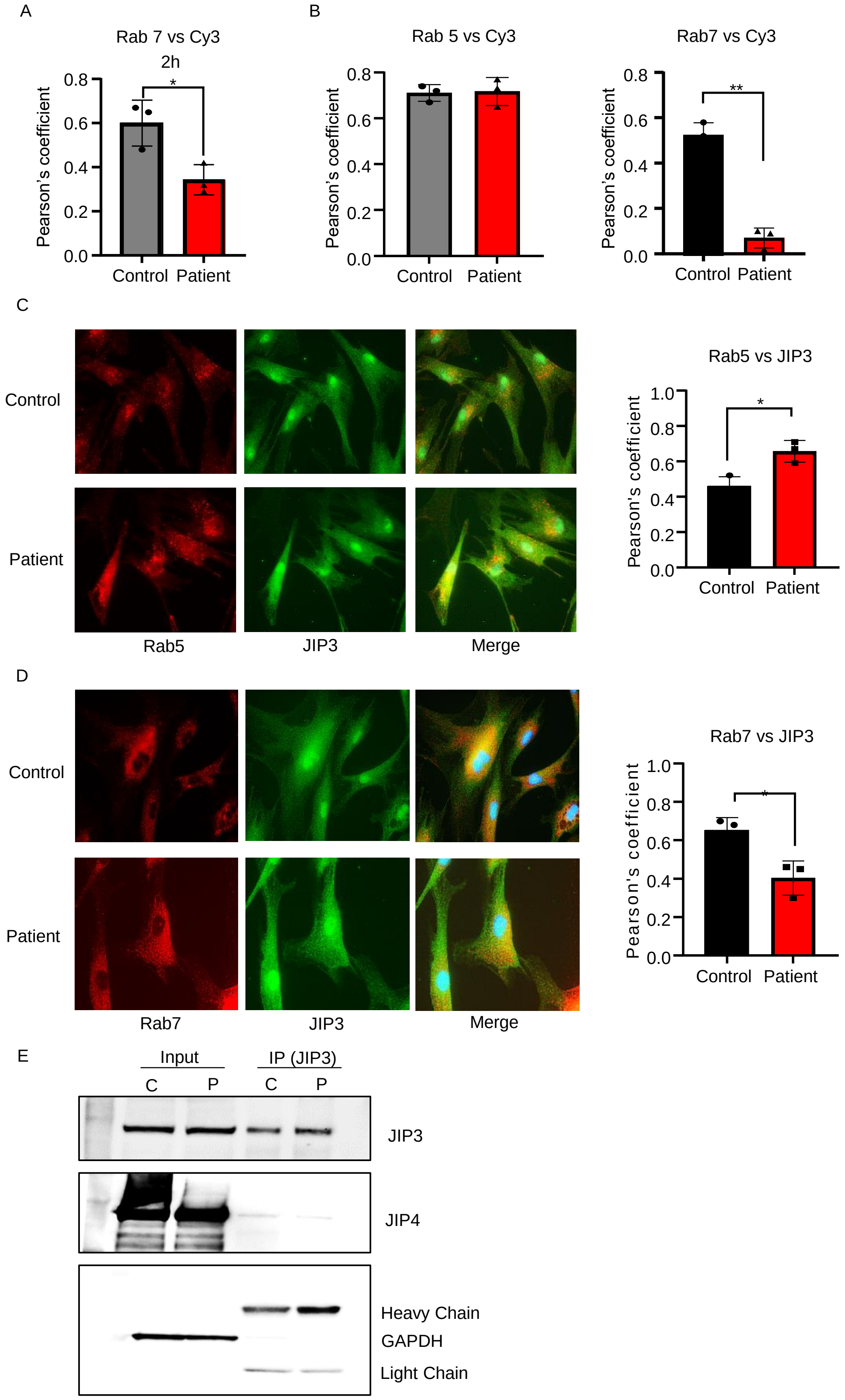

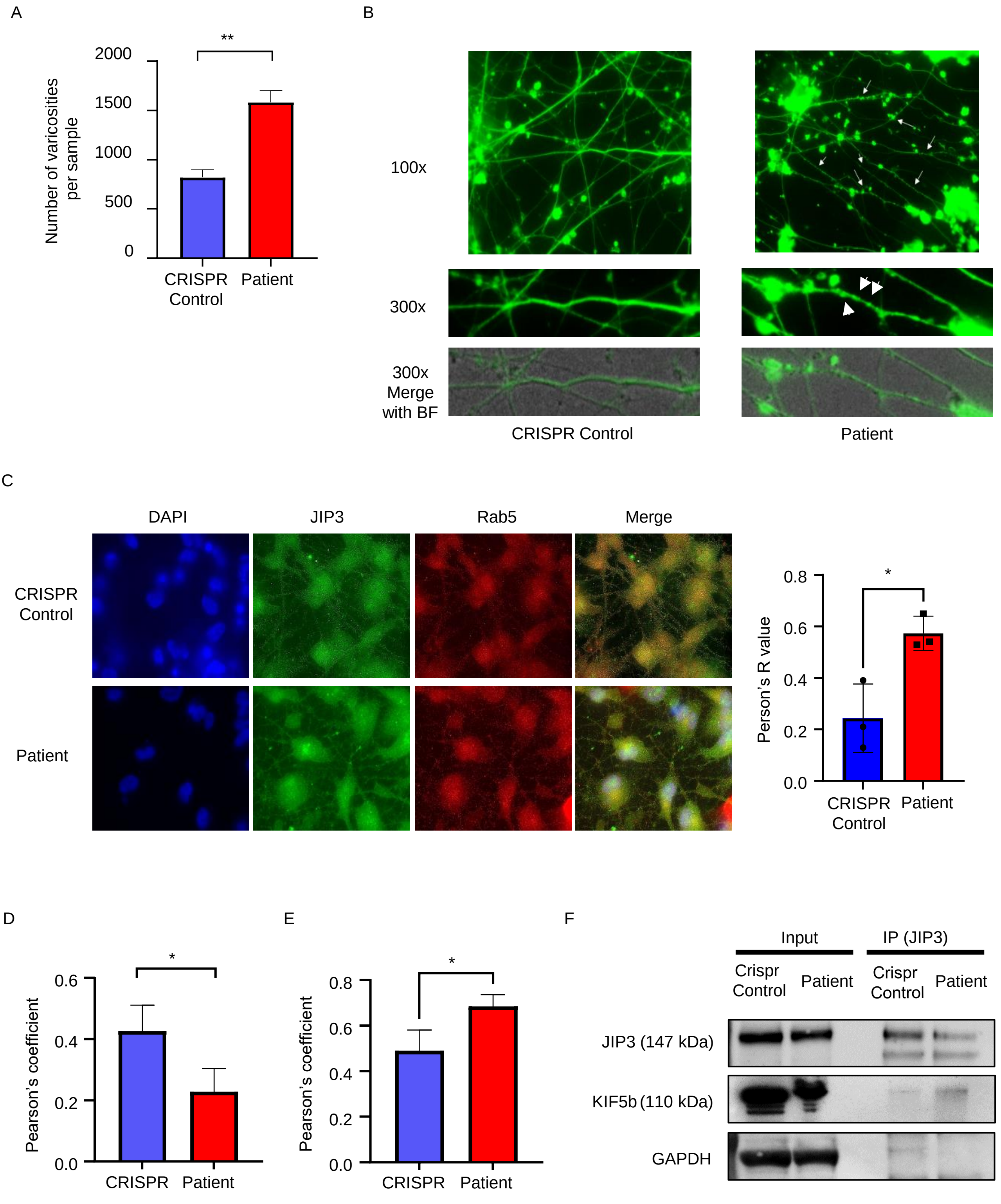
A
**
2000
1500
1000
500
0
Number of varicosities per sample
CRISPR Control
Patient
B
100x
300x
300x Merge with BF
CRISPR Control
Patient
C
DAPI
JIP3
Rab5
Merge
CRISPR Control
Patient
0.8
0.6
0.4
0.2
0.0
*
Person's R value
CRISPR Control
Patient
D
*
0.6
0.4
0.2
0.0
Pearson's coefficient
CRISPR
Patient
E
*
0.8
0.6
0.4
0.2
0.0
Pearson's coefficient
CRISPR
Patient
F
Input
IP (JIP3)
Crispr Control
Patient
Crispr Control
Patient
JIP3 (147 kDa)
KIF5b (110 kDa)
GAPDH

A

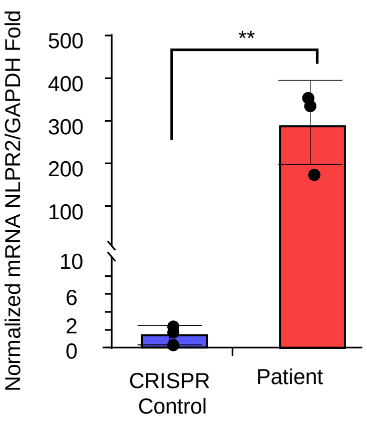
NLRP2
Normalized mRNA NLPR2/GAPDH Fold
500
400
300
200
100
10
6
2
0
**
CRISPR
Control
Patient

B

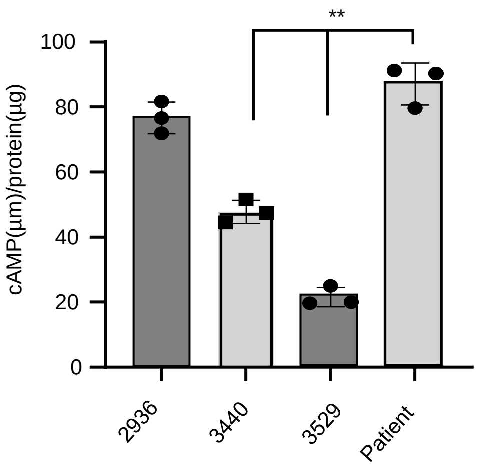
cAMP Level
cAMP(µm)/protein(µg)
100
80
60
40
20
0
**
2936
3440
3529
Patient

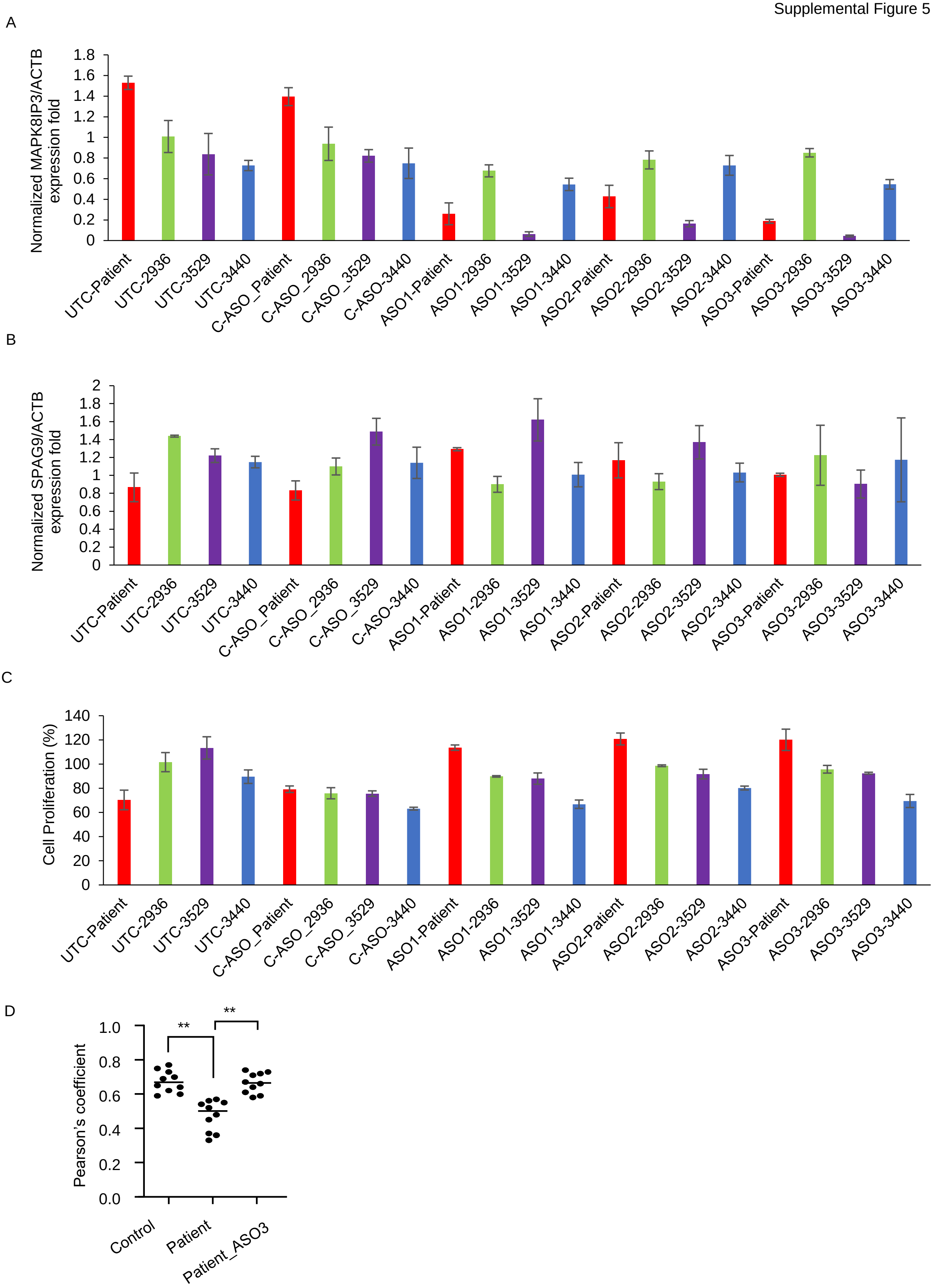
A
Normalized MAPK8IP3/ACTB expression fold
1.8
1.6
1.4
1.2
1
0.8
0.6
0.4
0.2
0
UTC-Patient
UTC-2936
UTC-3529
UTC-3440
C-ASO_Patient
C-ASO_2936
C-ASO_3529
C-ASO-3440
ASO1-Patient
ASO1-2936
ASO1-3529
ASO1-3440
ASO2-Patient
ASO2-2936
ASO2-3529
ASO2-3440
ASO3-Patient
ASO3-2936
ASO3-3529
ASO3-3440
B
Normalized SPAG9/ACTB expression fold
2
1.8
1.6
1.4
1.2
1
0.8
0.6
0.4
0.2
0
UTC-Patient
UTC-2936
UTC-3529
UTC-3440
C-ASO_Patient
C-ASO_2936
C-ASO_3529
C-ASO-3440
ASO1-Patient
ASO1-2936
ASO1-3529
ASO1-3440
ASO2-Patient
ASO2-2936
ASO2-3529
ASO2-3440
ASO3-Patient
ASO3-2936
ASO3-3529
ASO3-3440
C
Cell Proliferation (%)
140
120
100
80
60
40
20
0
UTC-Patient
UTC-2936
UTC-3529
UTC-3440
C-ASO_Patient
C-ASO_2936
C-ASO_3529
C-ASO-3440
ASO1-Patient
ASO1-2936
ASO1-3529
ASO1-3440
ASO2-Patient
ASO2-2936
ASO2-3529
ASO2-3440
ASO3-Patient
ASO3-2936
ASO3-3529
ASO3-3440
D
Pearson's coefficient
1.0
0.8
0.6
0.4
0.2
0.0
**
**
Control
Patient
Patient_ASO3

A

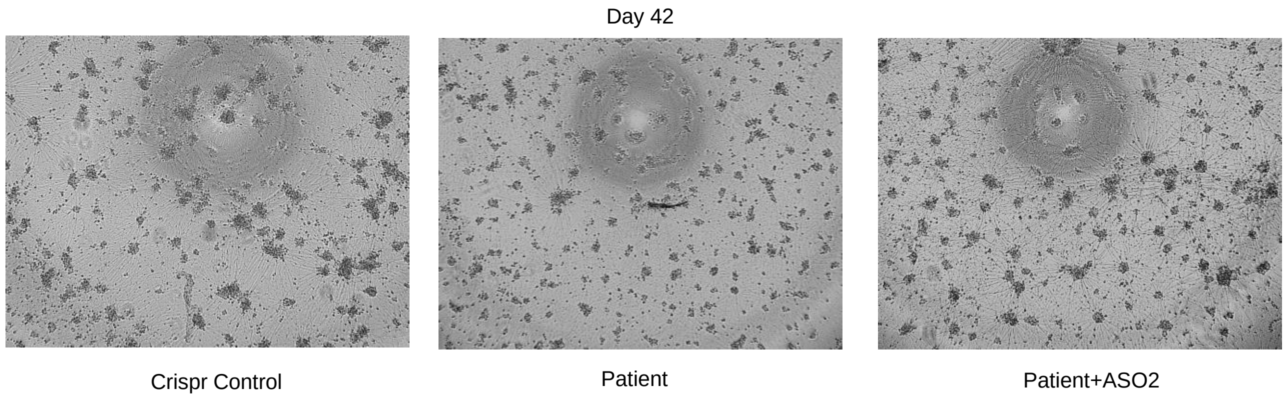
Day 42
Crispr Control
Patient
Patient+ASO2

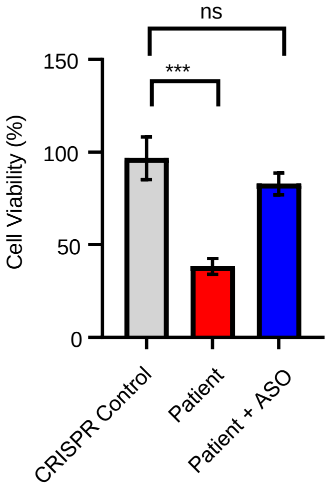
ns
***
150
100
50
0
Cell Viability (%)
CRISPR Control
Patient
Patient + ASO

Supplementary Table 1. (PPSets) Taqman assays

**Name**	**Target**	**Manufacturer**	**Cat#**
MAPKrs2076431	MAPK8IP3 Wt/Mt	ThermoFisher Scientific	4351379
Hs00187715_m1	SPAG9	ThermoFisher Scientific	4331182
Mm01301203_m1	MAPK8I3 (Ms)	ThermoFisher Scientific	4331182
Hs01049862_m1	MAPK8IP3 (Human)	ThermoFisher Scientific	4331182
Hs00265245_s1	DRD1	ThermoFisher Scientific	4331182
Hs00241436_m1	DRD2	ThermoFisher Scientific	4331182
Hs.PT.39a.22214847	ACTB	Integrated DNA technologies	-
siJIP4	JIP4	Santa Cruz Biotechnology	sc-62514
Control siRNA-A	N/A	Santa Cruz Biotechnology	sc-37007

Table S1. List of Primer probe sets and siRNAs used in the study, their manufacturer and catalogue number

Supplementary Table 2. (Antibodies)

Antibody	Host	Manufacturer	Cat#
JIP3 Polyclonal antibody	Rb	Proteintech	25212-1-AP
Phospho-c-Jun (Ser63) (54B3)	Rb	Cell Signaling Technology	2361S
SAPK/JNK	Rb	Cell Signaling Technology	9252S
Phospho-c-Jun (Ser73) (D47G9)	Rb	Cell Signaling Technology	3270S
Phospho-SAPK/JNK (Thr183/Tyr185) (81E11)	Rb	Cell Signaling Technology	4668S
c-Jun (60A8)	Rb	Cell Signaling Technology	9165S
Rab5 (C8B1)	Rb	Cell Signaling Technology	3547S
Cleaved Caspase-3 (Asp175) (5A1E)	Rb	Cell Signaling Technology	9664S
Caspase-3 (D3R6Y)	Rb	Cell Signaling Technology	14220S
D1DR/Dopamine Receptor D1	Ms	Santa Cruz Biotechnology	sc-33660
D2DR/Dopamine D2 Receptor	Ms	Santa Cruz Biotechnology	sc-5303
JIP-4 Antibody (H-8)	Ms	Santa Cruz Biotechnology	sc-271492
Rab 7 Antibody (B-3)	Ms	Santa Cruz Biotechnology	sc-376362
JIP-3 Antibody (F-6):	Ms	Santa Cruz Biotechnology	sc-46663
GAPDH Antibody (G-9)	Ms	Santa Cruz Biotechnology	sc-365062
Ku80 Antibody (EPR3468)	Rb	Abcam	ab80592
**Secondary Ab**	**Host**	**Manufacturer**	**Cat#**
Alexa Fluor™ 647	Rb	Invitrogen	A21245
Alexa Fluor™ 488	Ms	Invitrogen	A11001
Alexa Fluor™ 488	Ms	Invitrogen	A21042
Goat Anti-Rabbit	Rb	BioRad	1706515
Goat Anti-Mouse	Ms	BioRad	1706516

light chain specific Goat Anti-Mouse	Ms	Jackson Immuno Research Lab	115-035-174

Table S2. List of primary and secondary antibodies, host species, manufacturers and respective catalogue numbers.

Supplementary Table 3. (ASO)

ASO name	Target	Manufacturer	Sequence
1	MAPK8IP3	IDT	ACoAoGoAoTATATTTTAGCoGoAGT
2	MAPK8IP3	IDT	CCoAoGoToTTTTTCAGTAToToCCA
3	MAPK8IP3	IDT	ACoGoGoToCACATGGATGCoToCAC
Control	Non-targeting	IDT	CCoToAoTAGGACTATCCAoGoGoAA

Table S3. List of PS-ASOs and their respective targets and manufacturer details. Orange indicate 2'-MOE (2'-methoxyethyl) modifications; black represents a 2'-deoxyribosyl sugar moiety; 'o' means phosphodiester linkage; all other linkages are phosphorothioate.
